# Association of Lectin-Like Oxidized Low-Density Lipoprotein Receptor-1 With Angiotensin II Type 1 Receptor Impacts Mitochondrial Quality Control, Offering Promise for the Treatment of Vascular Senescence

**DOI:** 10.3389/fcvm.2021.788655

**Published:** 2021-11-17

**Authors:** Yoshihiro Uchikado, Yoshiyuki Ikeda, Yuichi Sasaki, Masaaki Iwabayashi, Yuichi Akasaki, Mitsuru Ohishi

**Affiliations:** Department of Cardiovascular Medicine and Hypertension, Graduate School of Medical and Dental Sciences Kagoshima University, Kagoshima, Japan

**Keywords:** mitochondrial dynamics, dynamin-related protein 1, mitochondrial autophagy, angiotensin II type 1 receptor, oxidized low-density lipoprotein, senescence

## Abstract

Lectin-like oxidized low-density lipoprotein (ox-LDL) causes vascular senescence and atherosclerosis. It has been reported that ox-LDL scavenger receptor-1 (LOX-1) is associated with the angiotensin II type 1 receptor (AT1R). While mitochondria play a crucial role in the development of vascular senescence and atherosclerosis, they also undergo quality control through mitochondrial dynamics and autophagy. The aim of this study was to investigate (1) whether LOX-1 associates with AT1R, (2) if this regulates mitochondrial quality control, and (3) whether AT1R inhibition using Candesartan might ameliorate ox-LDL-induced vascular senescence. We performed *in vitro* and *in vivo* experiments using vascular smooth muscle cells (VSMCs), and C57BL/6 and apolipoprotein E-deficient (ApoE KO) mice. Administration of oxidized low-density lipoprotein (ox-LDL) to VSMCs induced mitochondrial dysfunction and cellular senescence accompanied by excessive mitochondrial fission, due to the activation of fission factor Drp1, which was derived from the activation of the Raf/MEK/ERK pathway. Administration of either Drp1 inhibitor, mdivi-1, or AT1R blocker candesartan attenuated these alterations. Electron microscopy and immunohistochemistry of the co-localization of LAMP2 with TOMM20 signal showed that AT1R inhibition also increased mitochondrial autophagy, but this was not affected by Atg7 deficiency. Conversely, AT1R inhibition increased the co-localization of LAMP2 with Rab9 signal. Moreover, AT1R inhibition-induced mitochondrial autophagy was abolished by Rab9 deficiency, suggesting that AT1R signaling modulated mitochondrial autophagy derived from Rab9-dependent alternative autophagy. Inhibition of the Raf/MEK/ERK pathway also decreased the excessive mitochondrial fission, and Rab9-dependent mitochondrial autophagy, suggesting that AT1R signaling followed the Raf/MEK/ERK axis modulated both mitochondrial dynamics and autophagy. The degree of mitochondrial dysfunction, reactive oxygen species production, vascular senescence, atherosclerosis, and the number of fragmented mitochondria accompanied by Drp1 activation were all higher in ApoE KO mice than in C57BL/6 mice. These detrimental alterations were successfully restored, and mitochondrial autophagy was upregulated with the administration of candesartan to ApoE KO mice. The association of LOX-1 with AT1R was found to play a crucial role in regulating mitochondrial quality control, as cellular/vascular senescence is induced by ox-LDL, and AT1R inhibition improves the adverse effects of ox-LDL.

## Introduction

Hyperlipidemia is a major risk factor for atherosclerosis and senescence, and is reportedly related to cardiovascular disease (CVD), including coronary artery disease (1). There is increasing evidence that statins are effective for the primary and secondary prevention of CVD from a large clinical trial (2, 3). However, the incidence of CVD adverse events remains as high as 45%, even with statin treatment (4). Moreover, plaque progression is observed in one of three patients with lower levels of low-density lipoprotein (LDL) cholesterol (<70 mg/dl) in coronary artery lesions (5). Thus, the prevention of atherosclerotic CVD has a residual risk that is insufficient even if lipids are sufficiently lowered. Lectin-like ox-LDL scavenger receptor-1 (LOX-1) is a major receptor for ox-LDL and plays a crucial role in the genesis of reactive oxygen species (ROS) in vascular smooth muscle cells (VSMCs). It has recently been reported that LOX-1 associates with angiotensin II (Ang II) type 1 receptor (AT1R) and activates its downstream signal ERK in human umbilical vein endothelial cells (6, 7). The renin-angiotensin system (RAS), the main regulatory system for blood pressure, has been widely documented as being involved in the pathogenesis of several disease states, including CVD. RAS signaling was found to accelerate arterial senescence and arteriosclerosis in human VSMCs and an atherosclerotic apolipoprotein E-deficient (ApoE KO) mouse model (8, 9). Therefore, the treatment using AT1R blocker (ARB) based on the concept of association with LOX-1 and AT1R may have the potential to solve residual risk from lipid-lowering therapies in CVD patients. Indeed, several experimental and clinical studies revealed that ARB therapy combined with statin reduced atherosclerotic plaque of ApoE KO mice and improved clinical symptom and cardiac function in patients with CVD (10, 11).

Mitochondria, however, produce adenosine triphosphate (ATP), which generates ROS as a byproduct of ATP synthesis, and as their function declines, they yield more ROS, resulting in the development of vascular senescence and atherosclerosis. Thus, mitochondrial quality should be strictly controlled, as it can critically impact cellular viability and eventually an individuals' life. Mitochondria have a self-maintenance system to dynamically change their morphology, so-called mitochondrial dynamics (fission and fusion), and impaired mitochondria are eliminated through autophagy. Mitochondrial fission generates small spherical mitochondria, whereas fusion redistributes tubular or elongated mitochondria. Fusion is a mechanism that allows intact mitochondria to complement a damaged unit and share components, thereby maintaining metabolic efficiency. However, fission could isolate an altered subset of mitochondria and induce mitochondrial autophagy to eliminate damaged cells (12). Mitochondrial fission and fusion processes in mammals are mediated by several small GTPases, including dynamin-related protein 1 (Drp1), mitofusin 1 (Mfn1), mitofusin 2 (Mfn2), and optic atrophy 1 (Opa1) (13–15).

A previous study reported that AT1R KO mice had preserved mitochondrial function, leading to longevity (16). However, there are few reports on the relationship between the recovery of mitochondrial function from the effects AT1R inhibition and mitochondrial quality control through mitochondrial dynamics and autophagy. While the administration of ox-LDL decreased mitochondrial function in HL-1 cells, there is no report that this mitochondrial dysfunction is ameliorated by AT1R inhibition in terms of mitochondrial dynamics and autophagy (17). Therefore, we hypothesized that (1) the association of LOX-1 and AT1R is involved in mitochondrial quality control through mitochondrial dynamics and autophagy, and (2) AT1R inhibition using ARB, Candesartan, may ameliorate ox-LDL-mediated senescence, leading to atherosclerosis.

## Materials and Methods

### Cell Culture and Treatment

Human aortic smooth muscle cells were purchased from LONZA (Portsmouth, NH, USA) and cultured in smooth muscle basal medium-2 (Lonza, Cat #CC-3181) supplemented with SMC growth medium (Lonza, Cat #CC-4149), as described previously (18). Prior to stimulation, the cells were starved in 0.5 % FBS-containing medium for 16 h. The cells were treated with 20 μmol/L ox-LDL (Thermo Fisher Scientific, Cat #L34357). Time of the exposure to ox-LDL was 24 h for assessment of senescence in immunoblot (detections of p53 and p21) and in Senescence-associated β-galactosidase (SA-β gal) staining, 12 h for mitochondrial autophagy in immunohistochemistry, and 3 h for other experiments. The following inhibitors were used: 20 μmol/L Mdivi-1 (Sigma, Cat #M0199), Drp1 inhibitor; 10 μmol/L candesartan cilexetil (Candesartan) (Sigma-Aldrich, Cat #SML0245), ARB; 10 μmol/L Dabrafenib (Selleck, Cat #S2807), RAF inhibitor; 2 μmol/L PD184352 (Selleck, Cat #S1020), MEK inhibitor; 2 μmol/L SCH772984 (Selleck, Cat #S7101), ERK inhibitor. For the inhibition experiments, cells were treated with each inhibitor for 1 h following the addition of ox-LDL. Control cells were exposed to the same amount of dimethyl sulfoxide (DMSO). The dose of Candesartan was based on the previous report (19).

### Animal Models

This study was conducted in compliance with the protocol reviewed by the Institutional Animal Care and Use Committee and was approved by the Faculty of Medicine at Kagoshima University. All animal experiments followed the recommendations in the guidelines for animal experimentation at research institutes (Ministry of Education, Culture, Sports, Science and Technology, Japan), the guidelines for animal experimentation at institutes (Ministry of Health, Labor and Welfare, Japan), and the guidelines for proper conduct of animal experimentation (Science Council of Japan). In this experiment, we used only male mice, housed in groups of one to three per cage. Mice had free access to water and food during the experimental period and were maintained at room temperature (25 ± 2°C) under a standard 12/12 h light-dark cycle. For comparison of C57BL/6 and ApoE KO mice, ~35-week-old mice were used. To suppress mitochondrial fission, ApoE KO mice that were ~30 weeks old were administered either mdivi-1 (1.2 mg/kg) or with the same amount of DMSO by intraperitoneal injection for 4 weeks. To check the role of AT1R inhibition, ApoE KO mice at 16 weeks of age were administered either candesartan (10 mg/kg) or the same amount of DMSO by intraperitoneal injection for 8 weeks. Body weight and food intake were measured weekly after the injection. Systolic and diastolic blood pressure and heart rate of mouse were measured by MK-2000, a computer-controlled tail-cuff system (Muromachi Kikai).

### Tissue Preparation

All mice were sacrificed with an overdose of sodium pentobarbital (100 mg/kg) by intraperitoneal injection. ATP production, H_2_O_2_ measurement, and senescence-associated β-galactosidase (SA-β gal) staining were performed in a single mouse. For ATP production and H_2_O_2_ measurement, we isolated the mitochondrial fraction from vessels immediately after removal of aorta samples from the ascending aorta to the bifurcation of the common iliac artery. For SA-β gal staining, the aortic trees were removed and cut longitudinally from the ascending aorta to the abdominal aorta. We performed immunohistochemical analysis, Masson's trichrome staining and immunoblot analysis for the same mouse. For Masson's trichrome staining and immunohistochemical analysis, ascending aortas were fixed in 4% paraformaldehyde phosphate buffer solution, embedded in paraffin, and sectioned into 4 μm tissue sections. For immunoblot analysis, the remaining aorta samples from the ascending aorta to the bifurcation of the common iliac artery were rinsed with phosphate-buffered saline (PBS) and stored at −80°C.

### Immunoblot Analysis

Immunoblot analysis was performed in accordance with a previous report (18, 20). The primary antibodies used were as follows: Drp1 (1:1000, BD Biosciences, Cat #611112, RRID:AB_398423), Phospho-Drp1 ser616 (1:200, Cell Signaling Technology, Cat #4494, RRID:AB_11178659), Phospho-Drp1 ser637 (1:200, Cell Signaling Technology, Cat #4867, RRID:AB_10622027; 1:500, Abcam, Cat #ab193216), Opa1 (1:1000, BD Biosciences, Cat #612606, RRID:AB_399888), Mfn1 (1:1000, Abcam, Cat #ab57602, RRID:AB_2142624), Mfn2 (1:500, Sigma-Aldrich, Cat #M6319, RRID:AB_477221), COX IV (1:1000, Cell Signaling Technology, Cat #4844, RRID:AB_2085427), p53 (1:1000, Abcam, Cat #ab131442, RRID:AB_11155283; 1:200, Santa Cruz Biotechnology, Cat # sc-99, RRID:AB_628086), p21 (1:2000, Abcam, Cat #ab109199, RRID:AB_10861551), ERK1/2 (1:500, Santa Cruz Biotechnology, Cat #sc-514302, RRID:AB_2571739), Phospho-ERK1/2 (1:500, Santa Cruz Biotechnology, Cat #sc-7383, RRID:AB_627545), MEK1/2 (1:500, Santa Cruz Biotechnology, Cat #sc-81504, RRID:AB_1126111), Phospho-MEK1/2 (1:500, Santa Cruz Biotechnology, Cat #sc-81503, RRID:AB_1126112), CRAF (1:1000, Cell Signaling Technology, Cat #9422, RRID:AB_390808), Phospho-CRAF ser338 (1:500, Cell Signaling Technology, Cat #9427, RRID:AB_2067317), β-actin (1:1000, Santa Cruz Biotechnology, Cat #sc-47778 HRP, RRID:AB_2714189), and α-tubulin (1:1000, Sigma-Aldrich, Cat #T6199, RRID:AB_477583). The secondary antibodies used were as follows: goat anti-rabbit antibody (1:4000, Bio-Rad, Cat #170-6515, RRID:AB_11125142) or goat anti-mouse antibody (1:2000, Santa Cruz Biotechnology, Cat #sc-516102, RRID:AB_2687626). Bands were visualized using ECL Prime western blotting Detection Reagent (Amersham, Cat #RPN2232) and detected with Lumino Graph II (ATTO).

### Confocal Immunohistochemistry

Immunostaining *in vivo* and *in vitro* was performed as described previously (18, 21). The samples were stained with primary and secondary antibodies as follows: anti-Rab9 mouse monoclonal antibody (1:100, Abcam, Cat #ab2810, RRID:AB_303323), anti-LAMP2 rabbit polyclonal antibody (1:100, Sigma, Cat #L0668, RRID:AB_477154), anti-LAMP2 mouse monoclonal antibody (1:100, Abcam, Cat #ab25631, RRID:AB_470709), anti-TOMM20 rabbit monoclonal antibody (1:100, Abcam, Cat #ab186734, RRID:AB_2716623), anti- LC3B rabbit polyclonal antibody (1:100, Abcam, Cat #ab51520, RRID:AB_881429), Alexa Fluor 488-conjugated goat anti-rabbit IgG (1:100, Abcam, Cat #ab150081, RRID:AB_2734747), and Alexa Fluor 594-conjugated goat anti-mouse IgG (1:100, Abcam, Cat #ab150120, RRID:AB_2631447). Fluorescence images were obtained using an LSM700 confocal laser scanning microscope (Zeiss, Cat #LSM700, RRID:SCR_017377). Pearson's correlation coefficient was calculated to quantify the colocalization correlation of the intensity distributions between the two channels, as previously described (22). We performed independent *in-vitro* experiments three times.

### SA-β Gal Staining

SA-β gal activity in VSMCs was evaluated using the SA-β gal staining kit (Cell Signaling Technology, Cat #9860), according to the manufacturer's protocol. SA-β gal activity *in vitro* was assessed using β galactosidase staining solution (pH 6.0): 930 μl Staining solution, 10 μl solution A, 10 μl solution B, 50 μl DMF containing with 1 mg X-gal. Briefly, cells were washed with PBS and fixed with Fixative solution. Then they were stained with β galactosidase staining solution for 12 h at 37°C. SA-β gal positive cells were quantitatively determined within at least 200 cells that were counted in no less than four random fields using a microscope (Keyence, Cat #BZ X-710, RRID:SCR_017202). As described previously, SA-β gal activity *in vivo* was assessed using SA-β gal solution: 1 mg/ml X-Gal (Invitrogen, Cat #15520034), 1 mol/L MgCl_2_, 2% NP-40, 100 mmol/L potassium ferrocyanide (II), 100 mmol/L potassium ferrocyanide (III), and 40 mmol/L citric acid/sodium phosphate buffer (pH 6.0) (18). Briefly, after washing the harvested aortic trees from the ascending aorta to the abdominal aorta with PBS, they were stained with SA-β gal solution for 24 h at 37°C. The samples were then washed three times with PBS and fixed with 4% paraformaldehyde. Images were captured using a digital camera (Nikon, Cat #D810). SA-β gal positive area were quantified using ImageJ FIJI (National Institutes of Health, RRID:SCR_003070).

### Evaluation of Mitochondrial Reactive Oxygen Species and Membrane Potential Measurements

The methods for evaluation of mitochondrial reactive oxygen species and membrane potential measurements have been described previously (21). Briefly, intracellular mitochondrial ROS generation was measured using the MitoSox™ Red mitochondrial superoxide indicator (Invitrogen, Cat #M36008) under a microscope (Keyence, Cat #BZ-X710, RRID:SCR_017202). To evaluate mitochondrial membrane potential, the state of the mitochondrial membrane potential was visualized using MitoPT^®^JC-1 (ImmunoChemistry Technologies, Cat #924) with red and green fluorescence indicating polarized and depolarized mitochondria. Depolarized mitochondria were evaluated by counting the number of green fluorescent cells in at least 150 cells in each experiment.

### Mitotracker Staining

After administration of either ox-LDL treated with or without each inhibitor, or control vehicle to VSMCs as described above, the cells were stained with MitoTracker Red FM (Invitrogen, Cat #M22425), according to the manufacturer's protocol. Fluorescence images of live cells were acquired using a LSM700 confocal laser scanning microscope. The method for evaluating mitochondrial morphology has been described previously (21). More than 20 cells were chosen randomly in each experiment, and the percentage of cells with fragmented mitochondria was calculated.

### Mitochondrial Isolation

Mitochondrial fractions of VSMCs or isolated mouse aortas were purified using a different mitochondrial isolation kit (Thermo Fisher Scientific, Cat #89874; Sigma-Aldrich, Cat #MITOISO1), according to the manufacture's instruction. Briefly, *in vitro* experiment, cells were collected by cell scraper and then centrifuge at 850 × g for 2 min at 4°C. After removing supernatant samples were added several reagents and incubated according to the instruction. Then, samples were centrifuged at 700 × g for 5 min at 4°C. Supernatant were then centrifuged at 12,000 × g for 15 min at 4°C. After removing supernatant, the pellets were washed with 500 μl of Mitochondria Isolation Reagent C, and centrifuged again at 12,000 × g for 5 min at 4°C. The pellets contained mitochondria fraction. *In vivo* experiment, after washing the harvested aortic trees with Extraction Buffer A (EBA) [50 mmol/L HEPES, 1 mol/L mannitol, 350 mmol/L sucrose, 5 mmol/L EGTA], they were cut thinly and incubated in EBA containing 0.25 mg/ml trypsin on ice for 3 min. Then, removed supernatant and added 8 volumes of EBA containing 0.25 mg/ml trypsin for 20 min. After adding albumin solution and remove supernatant, samples were added eight volumes of EBA containing 0.25 mg/ml trypsin and homogenized. Homogenates were centrifuged at 600 × *g* for 5 min at 4°C. Supernatant were then centrifuged at 11,000 × *g* for 10 min at 4°C. The pellets were mitochondrial fractions. Regardless of *in vitro* or *vivo* experiments, performed all the isolation procedure at 4°C with ice-cold solutions.

### H_2_O_2_ Measurement and ATP Production

Mitochondrial fractions of cells and tissues were prepared as described above. H_2_O_2_ production was measured using an Amplex Red H_2_O_2_ assay kit (Invitrogen, Cat #A22188), according to the manufacturer's recommendations. ATP production was quantified using an ATP Bioluminescent Assay kit (Sigma-Aldrich, Cat #213-579-1), according to a previously described method (21).

### Masson's Trichrome Staining

We used 4 mice per group in comparison of C57BL/6 and ApoE KO mice, 3 mice per group in that of ApoE KO mice treated with mdivi-1 or the control, and 3 mice per group in that of ApoE KO mice treated with Candesartan or the control. Mice aorta samples were fixed as described above, and Masson's trichrome staining was used to visualize the state of fibrosis. Interstitial fibrosis was quantified using ImageJ FIJI (National Institutes of Health, RRID:SCR_003070).

### Transmission Electron Microscopy Analysis

The method of creating samples for EM has been described previously (21). Sections of the samples were observed using a HITACHI H-7650 transmission electron microscope (HITACHI).

### Evaluation of Average Mitochondrial Area

The number of mitochondria in each experiment was determined within at least 50 mitochondria, as assessed using EM. Mitochondria were randomly selected from no less than three independent experiments. The average mitochondrial area was calculated from the number of mitochondria and the total area of the mitochondria using ImageJ FIJI.

### Evaluation of Mitochondrial Autophagy

*In vitro* experiment, the cells of each group were quantitatively determined within at least 15 cells, counted in no less than three independent experiments assessed using EM. *In vivo* experiment, 600 μm^2^ of the aorta of each mouse was observed in a random fashion. The number of mitochondria engulfed by autophagosomes was counted using the EM. Three samples were assessed per group.

### Small Interfering RNA Transfection

For ERK1, ERK2, and Rab9 RNA knockdown, cells were transfected with ERK1, ERK2, Rab9, or control siRNA (Santa Cruz, Cat #sc-29307, sc-35335, sc-44065, sc-37007) using the siRNA transfection reagent (Santa Cruz, Cat #sc-29528), according to the manufacturer's protocol. For siRNA targeting *Atg7*, we transfected cells with *Atg7* or control (Thermo Fisher Scientific, Cat #s20650, #4390843) using the Lipofectamine RNAiMAX transfection reagent (Invitrogen™, 13778150), according to the manufacturer's protocol. Efficient knockdowns of ERK1, ERK2, Rab9, and *Atg7* were confirmed by immunoblotting (Supplementary Figures 1A–D).

### Statistical Analysis

All data are presented as mean ± standard error of the mean (SEM). For datasets with a small sample size (*n* = 3–5) or a biased distribution, the results were assessed statistically for significant differences by non-parametric statistical analysis using Wilcoxon rank sum test. Otherwise, statistical significance was assessed using Student's *t*-test. All statistical analyses were performed using the JMP Pro 15 (SAS Institute). Statistical significance was set at *P* < 0.05.

## Results

### ox-LDL Induced Mitochondrial Fission, ROS Production, Mitochondrial Dysfunction, and Cellular Senescence

**Figure 1:**
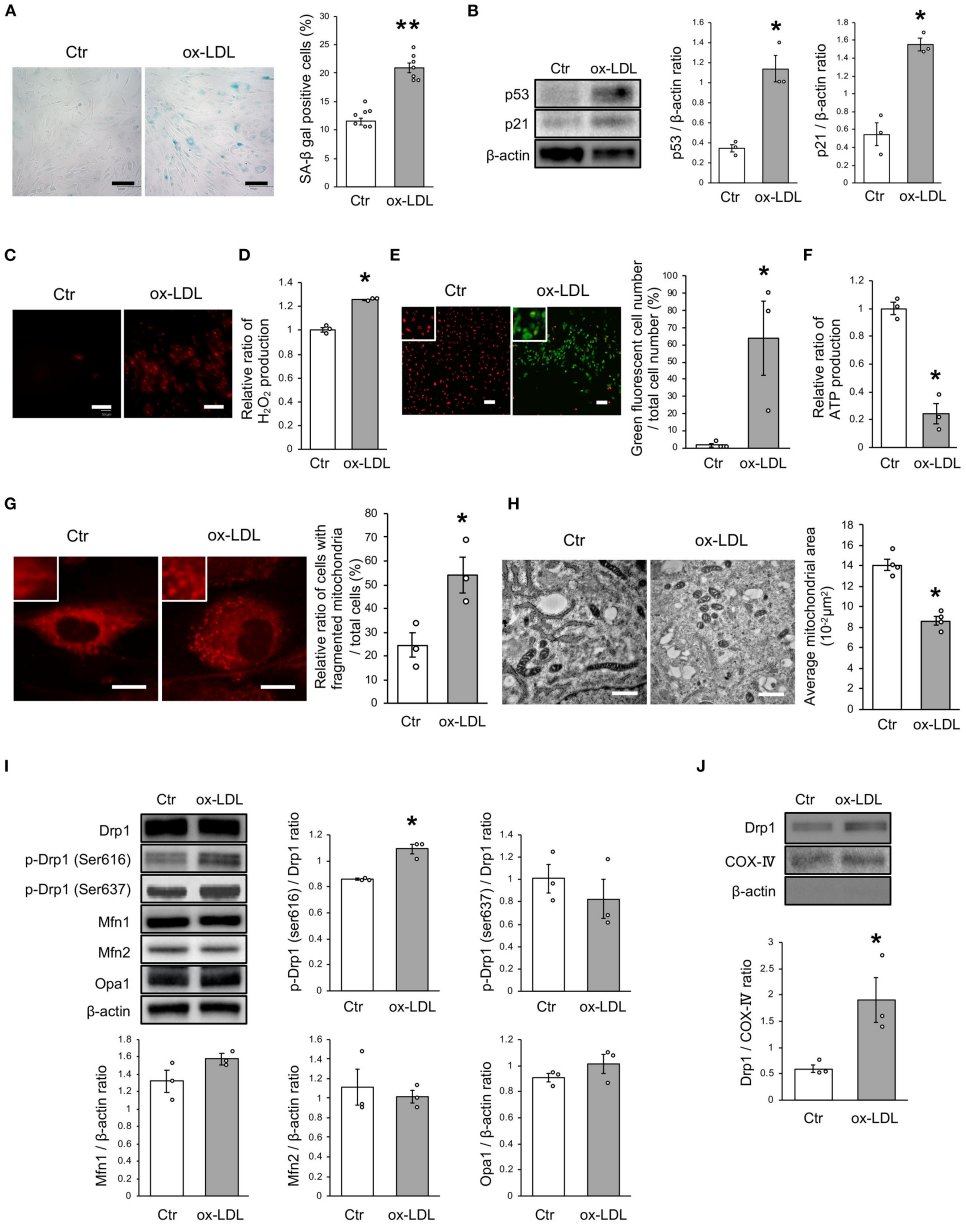
Ox-LDL treatment induced mitochondrial fission, ROS production, mitochondrial dysfunction, and cellular senescence. **(A)** Representative images and quantitative analysis of SA-β gal staining of cells treated with ox-LDL or the control. Scale bar = 100 μm. ^**^P < 0.01 vs. Ctr (*n* = 7 per group). **(B)** Immunoblots and quantitative analysis of p53, p21, and β-actin in cells treated with ox-LDL or the control. ^*^P < 0.05 vs. Ctr (*n* = 3 per group). **(C)** Representative images of MitoSox Red in cells treated with ox-LDL or the control. Scale bar = 100 μm. **(D)** Relative mitochondrial H_2_O_2_ production in cells treated with ox-LDL or the control. ^*^P < 0.05 vs. Ctr (*n* = 3 per group). **(E)** Representative images and quantitative analysis of JC-1 staining in cells treated with ox-LDL or the control. Scale bar = 100 μm. ^*^P < 0.05 vs. Ctr (*n* = 3 per group). **(F)** Relative mitochondrial ATP production in cells treated with ox-LDL or the control. ^*^P < 0.05 vs. Ctr (*n* = 3 per group). **(G)** Representative images of Mitotracker Red in cells treated with ox-LDL or control. Scale bar = 10 μm. ^*^P < 0.05 vs. Ctr (*n* = 3 per group). **(H)** Electron microscopic images and quantitative analysis of mitochondria and average mitochondrial areas in cells treated with ox-LDL or the control. Scale bar = 1 μm. ^*^P < 0.05 vs. Ctr (*n* = 4 per group). **(I)** Immunoblots and quantitative analysis for Drp1, p-Drp1 (Ser616), p-Drp1 (Ser637), Mfn1, Mfn2, Opa1, and β-actin in cells with ox-LDL or the control. ^*^P < 0.05 vs. Ctr (*n* = 3 per group). **(J)** Immunoblots and quantitative analysis for Drp1, COX IV, and β-actin in mitochondrial fractions of cells treated with ox-LDL or the control. ^*^P < 0.05 vs. Ctr (*n* = 3 per group). All data are presented as the mean ± SEM. Statistical analysis were conducted by Wilcoxon rank sum test **(B,D-J)** and Student's *t*-test **(A)**.

First, we investigated the effects of ox-LDL on cellular senescence. ox-LDL was found to increase the ratio of SA-β gal positive cells (Figure 1A). The expression of p53 and p21 was also higher in VSMCs treated with ox-LDL (Figure 1B), suggesting that ox-LDL induces cellular senescence. We then evaluated oxidative stress and mitochondrial function. The intensity of MitoSox Red fluorescence and H_2_O_2_ production were found to be higher in ox-LDL-treated cells (Figures 1C,D). Mitochondrial membrane potential and ATP production were lower in ox-LDL-treated cells (Figures 1E,F), suggesting that ox-LDL decreased mitochondrial function and increased ROS production. To assess whether ox-LDL modulates mitochondrial dynamics in VSMCs, we performed MitoTracker Red and electron microscopic analyses. Administration of ox-LDL increased the ratio of cells with fragmented mitochondria (Figure 1G). Both fused and small spherical mitochondria without ox-LDL were identified (Figure 1H). However, most of the mitochondria treated with ox-LDL showed a small spherical shape (Figure 1H). These results indicated that ox-LDL forced mitochondrial fission. We consequently investigated how mitochondrial fission was regulated in response to ox-LDL. Immunoblot analysis revealed that ox-LDL induced phosphorylation of Drp1 at serine 616, which is the activating form of Drp1 and the primary regulator of mitochondrial fission, but it did not modulate other mitochondrial dynamics factors, such as Mfn1, Mfn2, and Opa1, which induced mitochondrial fusion (Figure 1I). Although Drp1 is localized primarily in the cytosol, ox-LDL assembles Drp1 to mitochondria (Figure 1J). These results suggest that ox-LDL induces mitochondrial fission through Drp1 activation and may cause ROS production, mitochondrial dysfunction, and cellular senescence.

### Drp1 Inhibition Retarded Excessive Mitochondrial Fission, Mitochondrial Dysfunction, ROS Production, and Cellular Senescence

**Figure 2:**
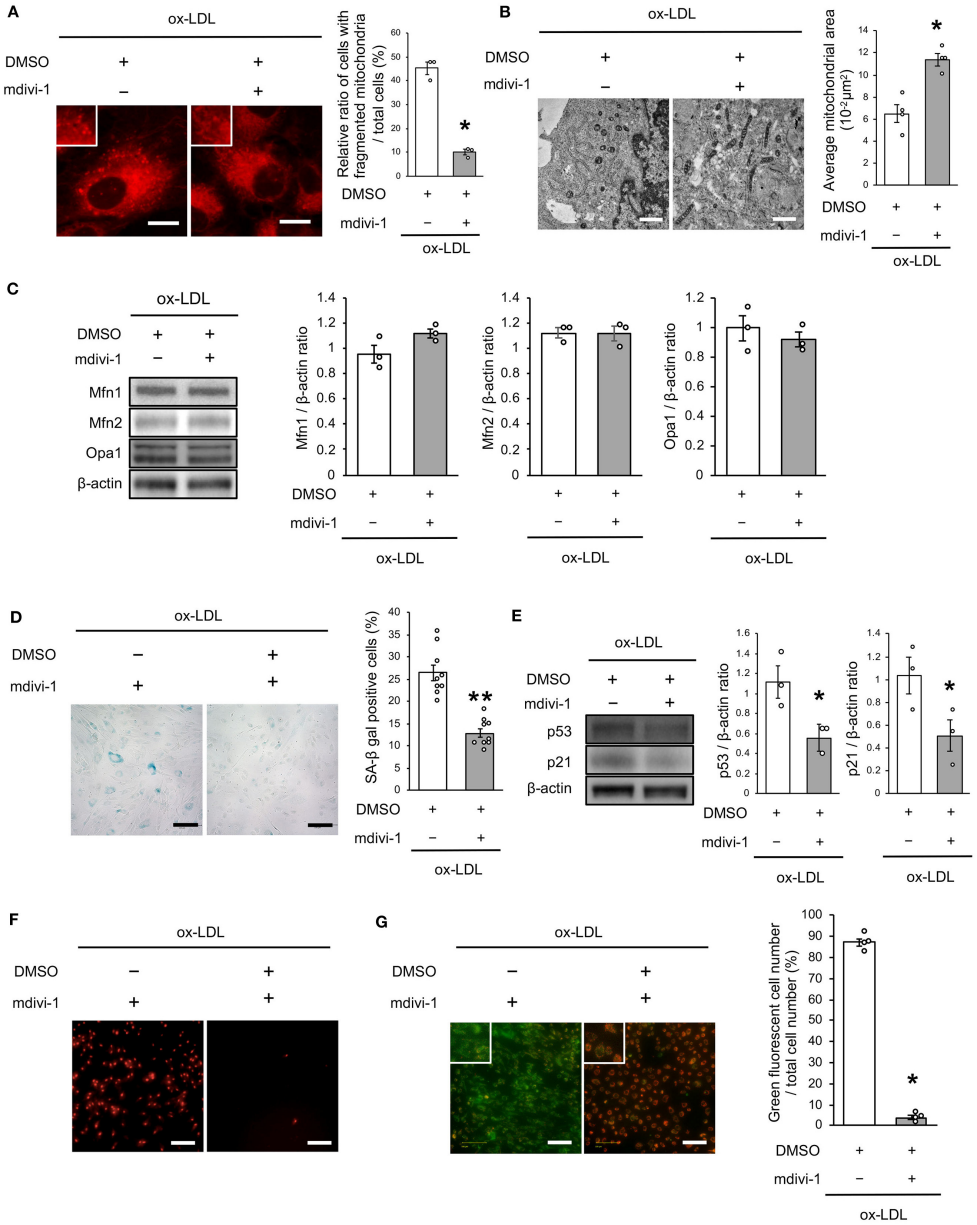
Mdivi-1 treatment retarded excessive mitochondrial fission, ROS production, mitochondrial dysfunction and cellular senescence, which were induced by ox-LDL. **(A)** Representative images and quantitative analysis of Mito-tracker Red in ox-LDL-treated cells with mdivi-1 or the control vehicle. Scale bar = 10 μm. ^*^P < 0.05 vs. ox-LDL with the control (*n* = 3 per group). **(B)** Electron microscopic images and quantitative analysis of mitochondria and average mitochondrial areas in ox-LDL-treated cells with mdivi-1 or the control. Scale bar = 1 μm. ^*^P < 0.05 vs. ox-LDL with the control (*n* = 4 per group). **(C)** Immunoblots and quantitative analysis for Mfn1, Mfn2, Opa1, and β-actin in ox-LDL-treated cells with mdivi-1 or the control (*n* = 3 per group). **(D)** Representative images and quantitative analysis of SA-β gal staining of ox-LDL-treated cells with mdivi-1 or the control. ^**^P < 0.01 vs. ox-LDL with the control (*n* = 9 per group). **(E)** Immunoblots and quantitative analysis for p53, p21, and β-actin in ox-LDL-treated cells with mdivi-1 or the control. ^*^P < 0.05 vs. ox-LDL with the control (*n* = 3 per group). **(F)** Representative images of MitoSox Red in ox-LDL-treated cells with mdivi-1 or the control. Scale bar = 100 μm. **(G)** Representative images and quantitative analysis of JC-1 staining in ox-LDL-treated cells with mdivi-1 or the control. Scale bar = 100 μm. ^*^P < 0.05 vs. ox-LDL with the control (*n* = 3 per group). All data are presented as the mean ± SEM. Statistical analysis were conducted by Wilcoxon rank sum test **(A-C,E,G)** and Student's *t*-test **(D)**.

To investigate whether ox-LDL-induced mitochondrial fission causes mitochondrial dysfunction, increased ROS production, and cellular senescence, we used mdivi-1, a small molecule that selectively inhibits the assembly of Drp1 to mitochondria. Mdivi-1 treatment decreased the ratio of cells with fragmented mitochondria induced by ox-LDL (Figure 2A). Moreover, mdivi-1 suppressed ox-LDL-mediated excessive mitochondrial fission and induced mitochondrial fusion (Figure 2B). However, mdivi-1 did not regulate mitochondrial fusion factors, such as Mfn1, Mfn2, and Opa1 (Figure 2C), indicating that mdivi-1 successfully restored mitochondrial fission by inhibiting Drp1. Under these conditions, mdivi-1 decreased the ratio of SA-β gal positive cells and the expression of p53 and p21 in VSMCs treated with ox-LDL (Figures 2D,E). The intensity of MitoSox Red fluorescence was lower in mdivi-1-treated cells than in untreated cells (Figure 2F). Furthermore, the administration of ox-LDL treated with mdivi-1 improved the mitochondrial membrane potential (Figure 2G). These results suggest that excessive mitochondrial fission is restored by Drp1 inhibition, mitochondrial dysfunction, ROS production, and cellular senescence.

### Receptor Association of LOX-1/AT1R Regulates Mitochondrial Dynamics

**Figure 3:**
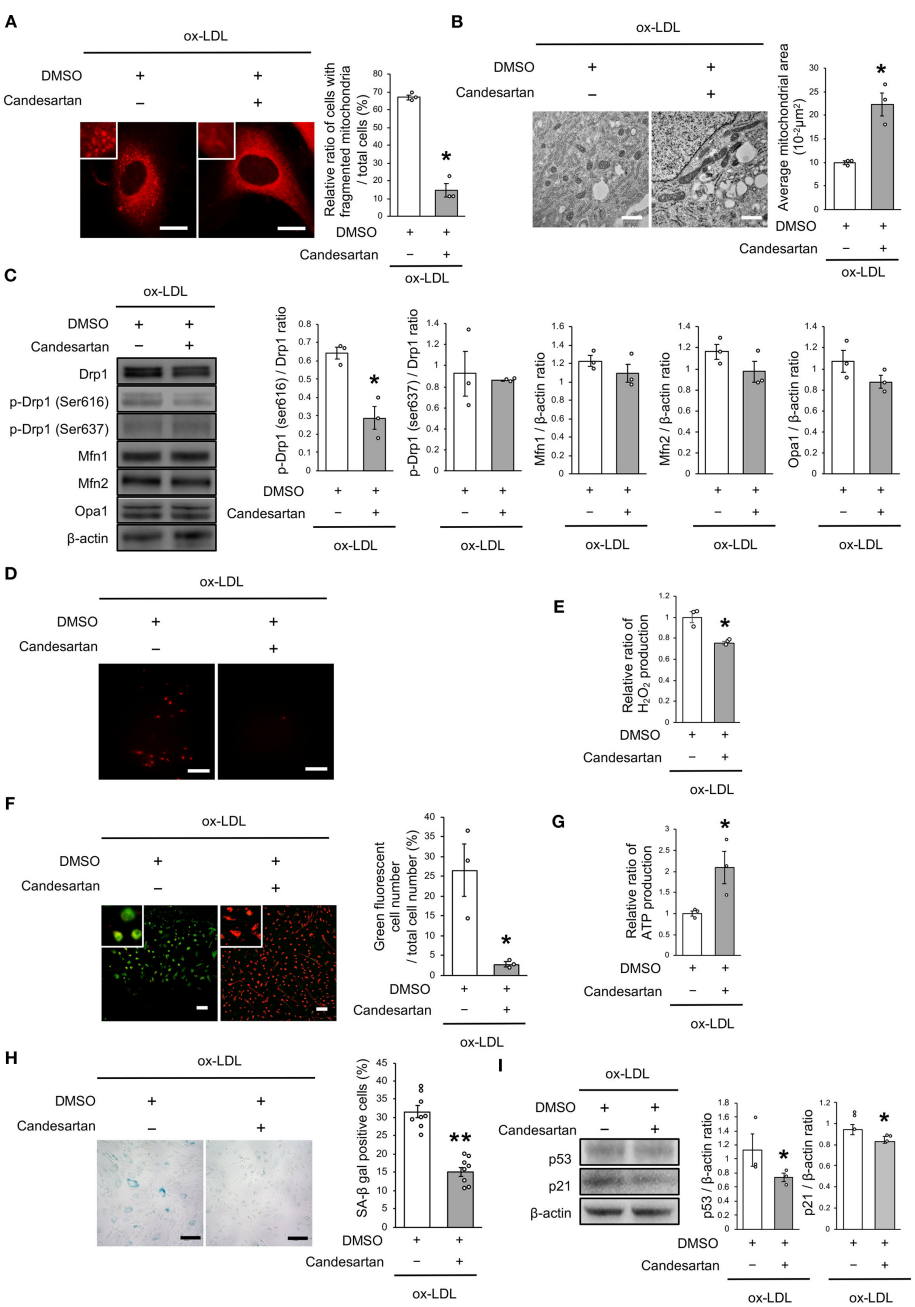
AT1R inhibition retarded excessive mitochondrial fission, ROS production, mitochondrial dysfunction, and cellular senescence, which are induced by ox-LDL. **(A)** Representative images and quantitative analysis of Mitotracker Red in ox-LDL-treated cells with Candesartan or the control vehicle. Scale bar = 10 μm. ^*^P < 0.05 vs. ox-LDL with the control (*n* = 3 per group). **(B)** Electron microscopic images and quantitative analysis of mitochondria and average mitochondrial areas in Candesartan-treated vs. vehicle-treated cells with ox-LDL. ^*^P < 0.05 vs. ox-LDL with the control (*n* = 3 per group). **(C)** Immunoblots and quantitative analysis for Drp1, p-Drp1 (Ser616), p-Drp1 (Ser637), Mfn1, Mfn2, Opa1, and β-actin in ox-LDL treated cells with Candesartan or the control. ^*^P < 0.05 vs. ox-LDL with the control (*n* = 3 per group). **(D)** Representative images of MitoSox Red in ox-LDL-treated cells with Candesartan or the control vehicle. **(E)** Relative mitochondrial H_2_O_2_ production in ox-LDL-treated cells with Candesartan or the control. ^*^P < 0.05 vs. ox-LDL with control vehicle (*n* = 3 per group). **(F)** Representative images and quantitative analysis of JC-1 staining in ox-LDL-treated cells with Candesartan or the control. Scale bar = 100 μm. ^*^P < 0.05 vs. ox-LDL with the control (*n* = 3 per group). **(G)** Relative mitochondrial ATP production in ox-LDL-treated cells with Candesartan or the control. ^*^P < 0.05 vs. ox-LDL with the control. (*n* = 3 per group). **(H)** Representative images and quantitative analysis of SA-β gal staining of ox-LDL-treated cells with Candesartan or the control. Scale bar = 100 μm. ^**^P < 0.01 vs. ox-LDL with the control (*n* = 8 per group). **(I)** Immunoblots and quantitative analysis for p53, p21, and β-actin in cells with Candesartan or the control under ox-LDL treatment. ^*^P < 0.05 vs. ox-LDL with the control (*n* = 3 per group). All data are presented as the mean ± SEM. Statistical analysis were conducted by Wilcoxon rank sum test **(A-C,E-G,I)** and Student's *t*-test **(H)**.

We next investigated whether the receptor association of LOX-1 with AT1R is involved in regulating mitochondrial dynamics using candesartan, an ARB. As shown in Figure 3A, AT1R inhibition decreased the ratio of cells with fragmented mitochondria (Figure 3A). Moreover, both fused and small spherical mitochondria were observed in VSMCs treated with candesartan (Figure 3B). Ox-LDL-induced Drp1 phosphorylation at Ser616 was also decreased by AT1R inhibition, but AT1R inhibition did not modulate other mitochondrial dynamics factors (Figure 3C). The intensity of MitoSox Red fluorescence and H_2_O_2_ production was lower in ARB-treated cells than in untreated cells (Figures 3D,E). Moreover, the administration of ox-LDL treated with ARB improved mitochondrial membrane potential and increased ATP production (Figures 3F,G). Furthermore, ARB decreased the ratio of SA-β gal positive cells and the expression of p53 and p21 in VSMCs treated with ox-LDL (Figures 3H,I). These results suggest that AT1R inhibition improved the imbalance of excessive mitochondrial fission and restored ROS production, mitochondrial dysfunction, and cellular senescence.

### ox-LDL Induced Drp1 Activation via the CRAF/MEK/ERK Pathway

**Figure 4:**
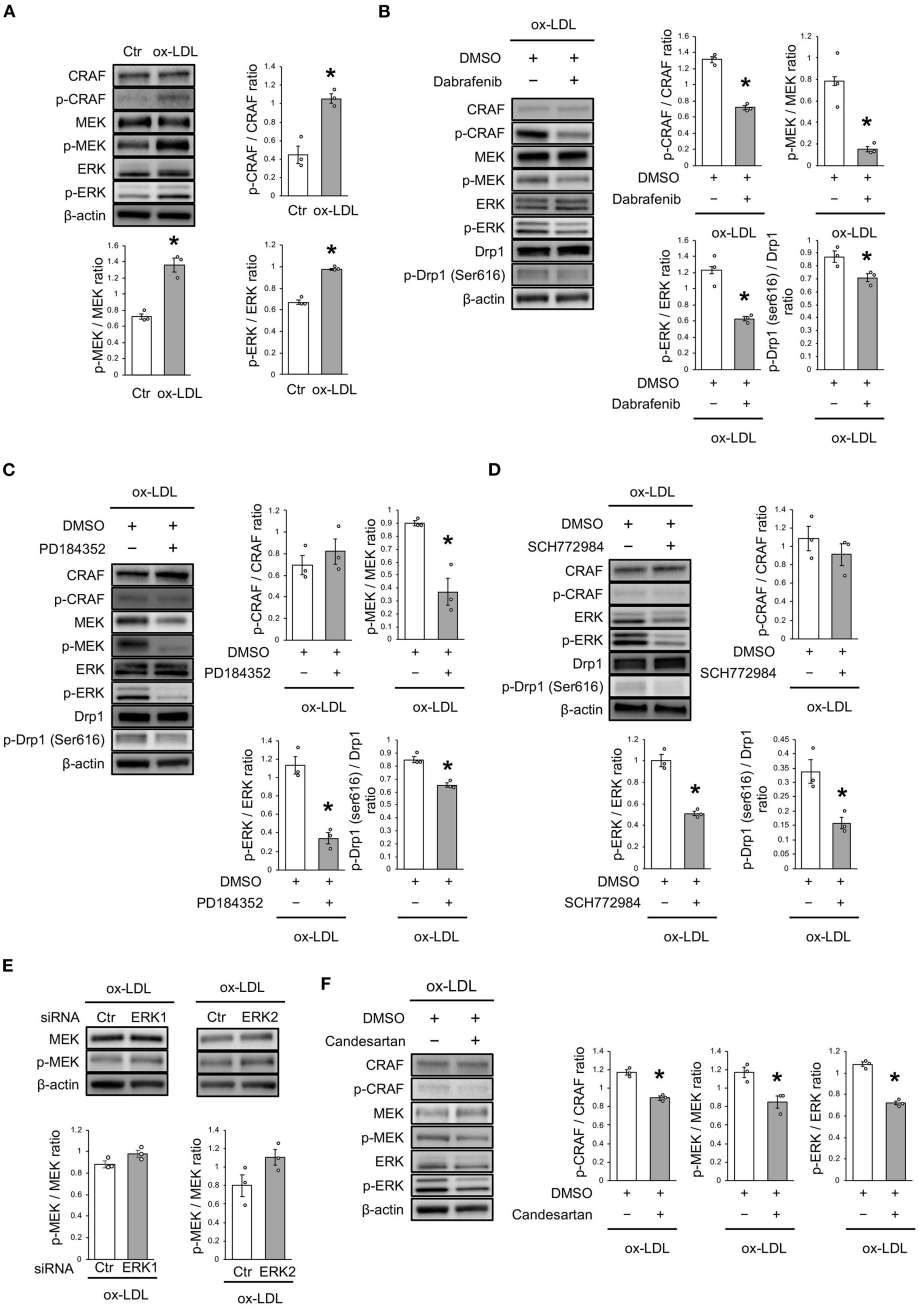
ox-LDL induced Drp1 activation through AT1R and the CRAF/MEK/ERK pathway. **(A)** Immunoblots and quantitative analysis for CRAF, p-CRAF, MEK, p-MEK, ERK, p-ERK, and β-actin in cells with ox-LDL or the control. ^*^P < 0.05 vs. the control (*n* = 3 per group). **(B)** Immunoblots and quantitative analysis for CRAF, p-CRAF, MEK, p-MEK, ERK, p-ERK, Drp1, p-Drp1 (Ser616), and β-actin in Dabrafenib-treated vs. control cells with ox-LDL. ^*^P < 0.05 vs. ox-LDL with the control (*n* = 3 per group). **(C)** Immunoblots and quantitative analysis for CRAF, p-CRAF, MEK, p-MEK, ERK, p-ERK, Drp1, p-Drp1 (Ser616), and β-actin in PD184352-treated vs. control cells with ox-LDL. ^*^P < 0.05 vs. ox-LDL with the control (*n* = 3 per group). **(D)** Immunoblots and quantitative analysis for CRAF, p-CRAF, MEK, p-MEK, ERK, p-ERK, Drp1, p-Drp1 (Ser616), and β-actin in SCH772984-treated vs. the control cells with ox-LDL. ^*^P < 0.05 vs. ox-LDL without SCH772984 (*n* = 3 per group). **(E)** Immunoblots and quantitative analysis for CRAF, p-CRAF, MEK, p-MEK, ERK, p-ERK, and β-actin in Candesartan-treated vs. control cells with ox-LDL. ^*^P < 0.05 vs. ox-LDL with the control (*n* = 3 per group). All data are presented as the mean ± SEM. Statistical analysis were conducted by Wilcoxon rank sum test **(A-F)**.

The mechanism of ox-LDL-induced mitochondrial fission was then investigated. Ox-LDL was found to activate CRAF/MEK/ERK, which is downstream of AT1R (Figure 4A). We consequently investigated whether CRAF, MEK, or ERK modulated Drp1 activation using inhibition experiments. The results showed that Dabrafenib, a RAF inhibitor, inactivated MEK, ERK, and Drp1 (Figure 4B). MEK inhibition using PD184352 inactivated ERK and Drp1, but not CRAF (Figure 4C). ERK inhibition using SCH772984 also inactivated Drp1, but not CRAF (Figure 4D). Knockdowns of ERK1 or ERK2 did not modulate activation of MEK (Figure 4E and Supplementary Figures 1A,B). The CRAF/MEK/ERK pathway was found to play a role in activating Drp1 (Figures 4D,E), while ARB decreased ox-LDL-induced CRAF/MEK/ERK activation (Figure 4F). These results suggest that ox-LDL activates Drp1 through CRAF/MEK/ERK signaling, and this is downstream of AT1R.

### AT1R Inhibition Induced Mitochondrial Autophagy Derived From Rab9-Dependent Alternative Autophagy

**Figure 5:**
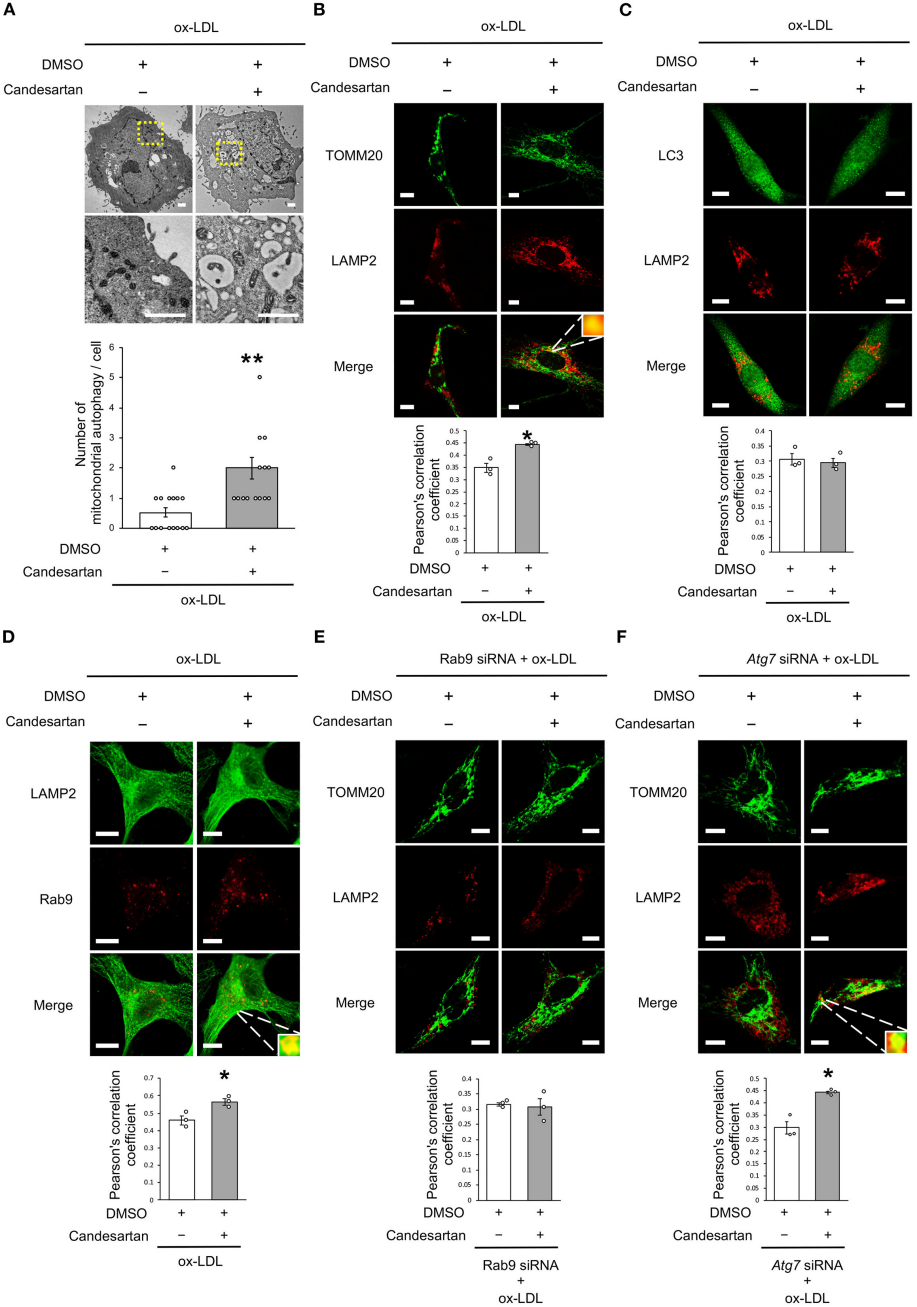
AT1R inhibition induced mitochondrial autophagy derived from Rab9-dependent alternative autophagy with ox-LDL treatment. **(A)** Electron microscopic images and quantitative analysis of the number of mitochondrial autophagy in Candesartan-treated vs. the control cells with ox-LDL. Scale bar = 1 μm. ^**^P < 0.01 vs. ox-LDL with the control (*n* = 15 per group). **(B)** Representative images and quantitative analysis of TOMM20 (green) and LAMP2 (red) immunohistochemistry in Candesartan-treated vs. control cells with ox-LDL. Scale bar = 10 μm. ^*^P < 0.05 vs. ox-LDL with the control (*n* = 3 per group). **(C)** Representative images and quantitative analysis of LC3 (green) and LAMP2 (red) immunohistochemistry in Candesartan-treated vs. control vehicle-treated cells with ox-LDL (*n* = 3 per group). Scale bar = 10 μm. **(D)** Representative images and quantitative analysis of LAMP2 (green) and Rab9 (red) immunohistochemistry in Candesartan-treated vs. control cells with ox-LDL. Scale bar = 10 μm. ^*^P < 0.05 vs. ox-LDL with the control (*n* = 3 per group). **(E)** Representative images and quantitative analysis of TOMM20 (green) and LAMP2 (red) immunohistochemistry in Candesartan-treated vs. control cells transfected with siRab9 under ox-LDL treatment. Scale bar = 10 μm. (*n* = 3 per group). **(F)** Representative images and quantitative analysis of TOMM20 (green) and LAMP2 (red) immunohistochemistry in Candesartan-treated vs. control cells transfected with si*Atg7* under ox-LDL treatment. Scale bar = 10 μm. ^*^P < 0.05 when compared with control vehicle-treated cells transfected with si*Atg7* under ox-LDL treatment (*n* = 3 per group). All data are presented as the mean ± SEM. Statistical analysis were conducted by Wilcoxon rank sum test **(B,C,E,F)** and Student's *t*-test **(A)**.

As dysfunctional mitochondria are eliminated through autophagy, which is generally believed to require mitochondrial fission, we investigated whether AT1R inhibition regulates mitochondrial autophagy. Electron microscopic analysis showed that mitochondrial autophagy increased in ox-LDL and ARB-treated cells (Figure 5A). Under ox-LDL treatment, immunohistochemistry analysis showed that the colocalization of mitochondrial protein TOMM20 and LAMP2, which is a marker of autolysosomes, was higher in ARB-treated cells than in untreated cells (Figure 5B). These results suggest that AT1R inhibition promotes mitochondrial autophagy. In contrast, ARB treatment did not change the colocalization of LAMP2 with LC3, which is a hallmark of autophagy (Figure 5C). Recent studies have revealed the existence of at least two forms of autophagy. One is LC3- and *Atg7*-dependent autophagy, which is also considered to be conventional autophagy. The other is called alternative autophagy, does not require the use of LC3 or *Atg7* but does require the membrane trafficking protein Rab9 (23). To examine whether mitochondrial autophagy is related to conventional autophagy or alternative autophagy, we examined the colocalization of LAMP2 with Rab9 using double-staining immunohistochemistry. The colocalization of LAMP2 with Rab9 staining was also significantly increased in ox-LDL-treated cells with ARB (Figure 5D). In addition, the colocalization of TOMM20 with LAMP2 induced by AT1R inhibition was not affected by *Atg7* knockdown, but by Rab9 knockdown in ox-LDL-treated cells (Figures 5E,F). Furthermore, Rab9 knockdown increased the ratio of SA-β gal positive cells in ox-LDL-treated cells with candesartan, indicating that AT1R inhibition induces mitochondrial autophagy through Rab9 (Supplementary Figure 1E). These results suggest that AT1R inhibition induces mitochondrial autophagy derived from Rab9-dependent alternative autophagy, leading to the suppression of cellular senescence by maintaining mitochondrial quality control.

### Rab9-Dependent Mitochondrial Autophagy Was Not Affected by ERK Inhibition but by MEK Inhibition

**Figure 6:**
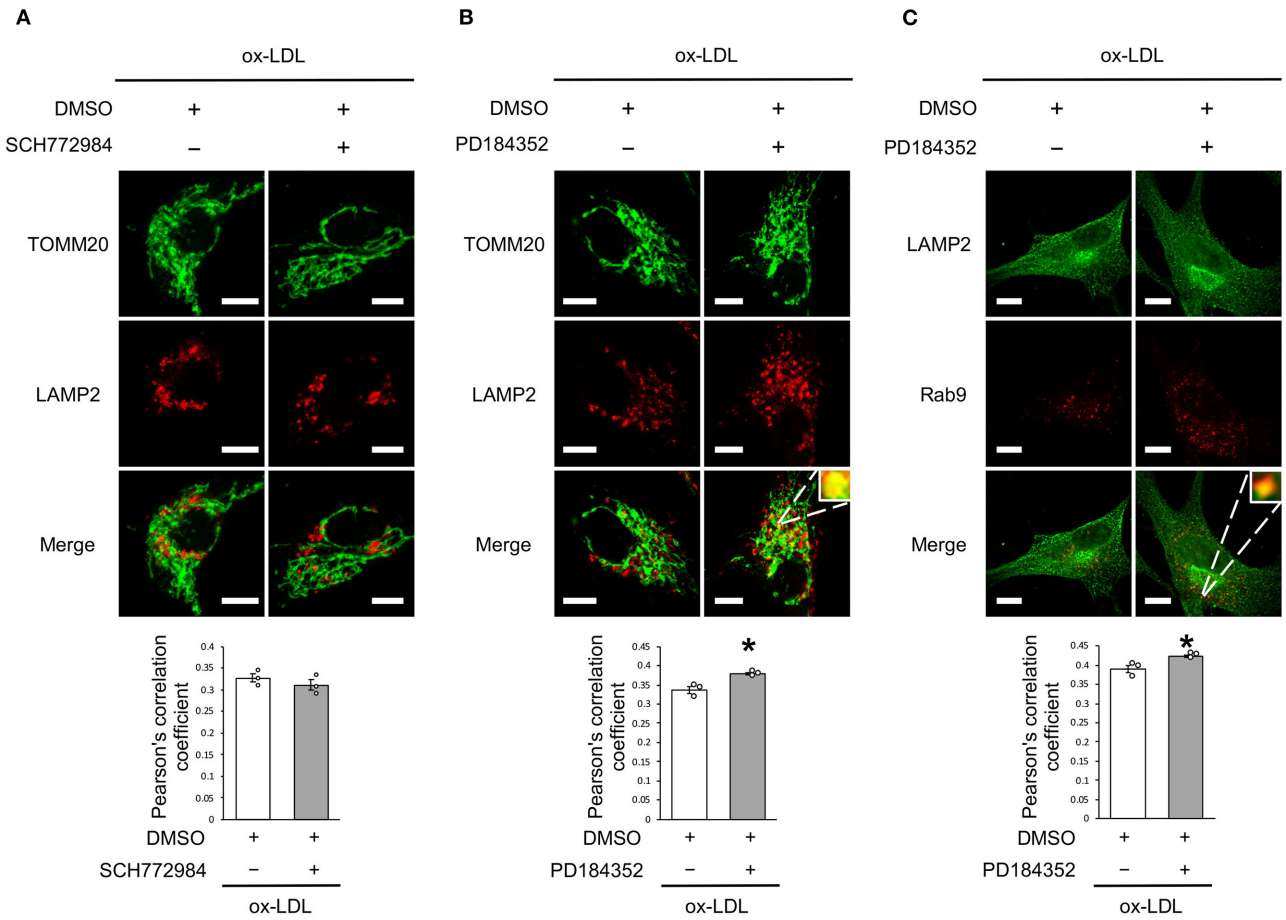
Rab9-dependent mitochondrial autophagy was not affected by ERK inhibition but was affected by MEK inhibition. **(A)** Representative images and quantitative analysis of TOMM20 (green) and LAMP2 (red) immunohistochemistry in SCH772984-treated vs. control vehicle-treated cells with ox-LDL. Scale bar = 10 μm. (*n* = 3 per group). **(B)** Representative images and quantitative analysis of TOMM20 (green) and LAMP2 (red) immunohistochemistry in PD184352-treated vs. control vehicle-treated cells with ox-LDL. Scale bar = 10 μm. ^*^P < 0.05 when compared with ox-LDL with the control (*n* = 3 per group). **(C)** Representative images and quantitative analysis of LAMP2 (green) and Rab9 (red) immunohistochemistry in PD184352-treated vs. control cells with ox-LDL. Scale bar = 10 μm. ^*^P < 0.05 when compared with ox-LDL with the control (*n* = 3 per group). All data are presented as the mean ± SEM. Statistical analysis were conducted by Wilcoxon rank sum test **(A-C)**.

We further analyzed whether CRAF/MEK/ERK is involved in AT1R inhibition-mediated mitochondrial autophagy derived from Rab9-dependent alternative autophagy. ERK inhibition did not affect the colocalization of LAMP2 and TOMM20 (Figure 6A), whereas MEK inhibition increased the colocalization of LAMP2 with either TOMM20 or Rab9 (Figures 6B,C), indicating that AT1R inhibition-induced mitochondrial autophagy is mediated by the AT1R/CRAF/MEK pathway.

### Drp1 Inhibition Improved the Imbalance of Mitochondrial Dynamics and Vascular Senescence in ApoE KO Mice

**Figure 7:**
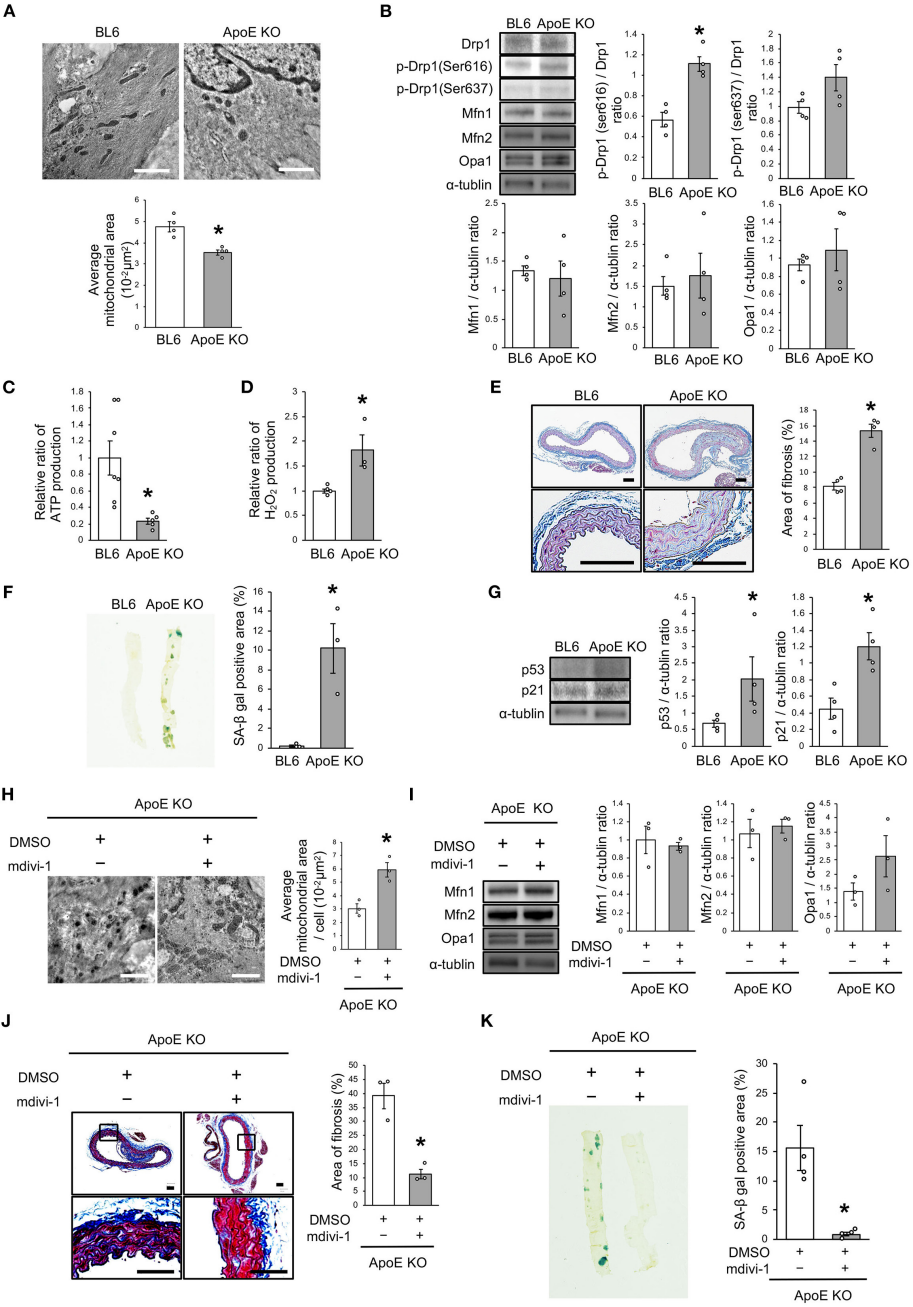
Drp1 inhibition improved the imbalance of excessive mitochondrial fission and vascular senescence in ApoE KO mice. **(A)** Electron microscopic images and quantitative analysis of mitochondria and average mitochondrial areas in C57BL/6 and ApoE KO mice. Scale bar = 1 μm. ^*^P < 0.05 vs. C57BL/6 (*n* = 4 per group). **(B)** Immunoblots and quantitative analysis for Drp1, p-Drp1 (Ser616), p-Drp1 (Ser637), Mfn1, Mfn2, Opa1, and α-tublin in C57BL/6 and ApoE KO mice. ^*^P < 0.05 vs. C57BL/6 (*n* = 4 per group). **(C)** Relative mitochondrial ATP production in C57BL/6 and ApoE KO mice. ^*^P < 0.05 vs. C57BL/6 (C57BL/6; *n* = 7, ApoE KO mice; *n* = 5). **(D)** Relative mitochondrial H_2_O_2_ production in C57BL/6 and ApoE KO mice. ^*^P < 0.05 vs. C57BL/6 (*n* = 4 per group). **(E)** Representative images and quantitative analysis of Masson's trichrome staining in C57BL/6 and ApoE KO mice. Scale bar = 100 μm. ^*^P < 0.05 vs. C57BL/6 (*n* = 4 per group). **(F)** Representative images and quantitative analysis of SA-β gal staining of arteries in C57BL/6 and ApoE KO mice. ^*^P < 0.05 vs. C57BL/6 (*n* = 3 per group). **(G)** Immunoblots and quantitative analysis for p53, p21, and α-tublin in C57BL/6 and ApoE KO mice. ^*^P < 0.05 vs. C57BL/6 (*n* = 4 per group). **(H)** Electron microscopic images and quantitative analysis of mitochondria and average mitochondrial areas in ApoE KO mice treated with mdivi-1 or the control. Scale bar = 1 μm. ^*^P < 0.05 vs. ApoE KO mice with the control (*n* = 3 per group). **(I)** Immunoblots and quantitative analysis for Mfn1, Mfn2, Opa1, and α-tublin in ApoE KO mice treated with mdivi-1 or the control, (*n* = 3 per group). **(J)** Representative images and quantitative analysis of Masson's trichrome staining in ApoE KO mice treated with mdivi-1 or the control. Scale bar = 100 μm. ^*^P < 0.05 vs. ApoE KO mice with the control (*n* = 3 per group). **(K)** Representative images and quantitative analysis of SA-β gal staining of the arteries in ApoE KO mice treated with mdivi-1 or the control. ^*^P < 0.05 vs. ApoE KO mice with the control (*n* = 3 per group). All data are presented as the mean ± SEM. Statistical analysis were conducted by Wilcoxon rank sum test **(A,B,D-K)** and Student's *t*-test **(C)**.

We investigated the relationship between vascular senescence and mitochondrial dynamics in C57BL/6 and ApoE KO mice. Body weight, heart rate, and blood pressure were not found to differ between C57BL/6 and ApoE KO mice (Table 1). EM revealed that ApoE KO mice forced mitochondrial dynamics resulting in excessive fission when compared to C57BL/6 mice (Figure 7A). Immunoblot analysis indicated that the arterial expression of Drp1 phosphorylation at serine 616 was higher in ApoE KO mice than in C57BL/6 mice, but other mitochondrial dynamics factors were not significantly different between the two groups (Figure 7B). ATP production was lower and H_2_O_2_ production was higher in ApoE KO mice than in C57BL/6 mice, suggesting that ApoE KO mice had reduced mitochondrial function and increased oxidative stress in the arteries (Figures 7C,D). Arterial fibrosis, was assessed using Masson's trichrome staining, and was found to have increased in ApoE KO mice when compared with that in C57BL/6 mice (Figure 7E). The ratio of arterial senescence assessed by SA-β gal staining was greater in ApoE KO mice than in C57BL/6 mice (Figure 7F). Immunoblot analysis showed that the expression of p21 and p53 was higher in ApoE KO mice than in C57BL/6 mice (Figure 7G). These results indicate that arterial senescence is facilitated in ApoE-KO mice. Next, we investigated ApoE KO mice treated with mdivi-1 to investigate whether the inhibition of mitochondrial fission retarded vascular senescence. Food intake during the experiment, body weight, heart rate, and blood pressure were not different between ApoE KO mice treated with mdivi-1 and the control vehicle (Table 2). EM analysis revealed that excessive mitochondrial fission was reduced in ApoE KO mice treated with mdivi-1 compared to those treated with the control vehicle (Figure 7H). Immunoblot analysis showed that the administration of mdivi-1 did not significantly change the expression of mitochondrial fusion factors (Figure 7I). The ratio of the arterial fibrosis and senescence area, assessed using Masson trichrome analysis and SA-β gal staining, respectively, were lower in ApoE KO mice administered mdivi-1 than in those treated with the control vehicle (Figures 7J,K). These results suggest that excessive mitochondrial fission through Drp1 activation promotes arterial senescence in ApoE KO mice.

**Table 1:** Body weight, heart rate, and blood pressure of the C57BL/6 and ApoE KO mice.

	**C57BL/6 (*n* = 18)**	**ApoE KO (*n* = 15)**	***P*** **-value**
BW (g)	32.8 ± 0.7	32.1 ± 0.5	N.S.
HR (bpm)	705 ± 11	668 ± 13	N.S.
BP (mmHg)	107 ± 4/63 ± 3	116 ± 5/71 ± 4	N.S.

*All values are presented as the mean and SD. BW, body weight; HR, heart rate; BP, blood pressure*.

**Table 2:** Body weight, food intake, heart rate, and blood pressure of ApoE KO mice administered with mdivi-1 or the control vehicle.

	**ApoE KO + control vehicle (*n* = 7)**	**ApoE KO + mdivi-1 (*n* = 7)**	***P*** **-value**
BW (g)	31.9 ± 1.3	31.4 ± 2.5	N.S.
FI (g/day)	3.7 ± 0.3	3.7 ± 0.4	N.S.
HR (bpm)	692 ± 35	657 ± 72	N.S.
BP (mmHg)	113 ± 18/76 ± 9	118 ± 16/72 ± 22	N.S.

*All values are presented as the mean and SD. BW, body weight; FI, food intake; HR, heart rate; BP, blood pressure*.

### AT1R Inhibition Improved the Imbalance of Mitochondrial Dynamics and Induced Rab9-Dependent Mitochondrial Autophagy, Leading to Anti-vascular Senescence

**Figure 8:**
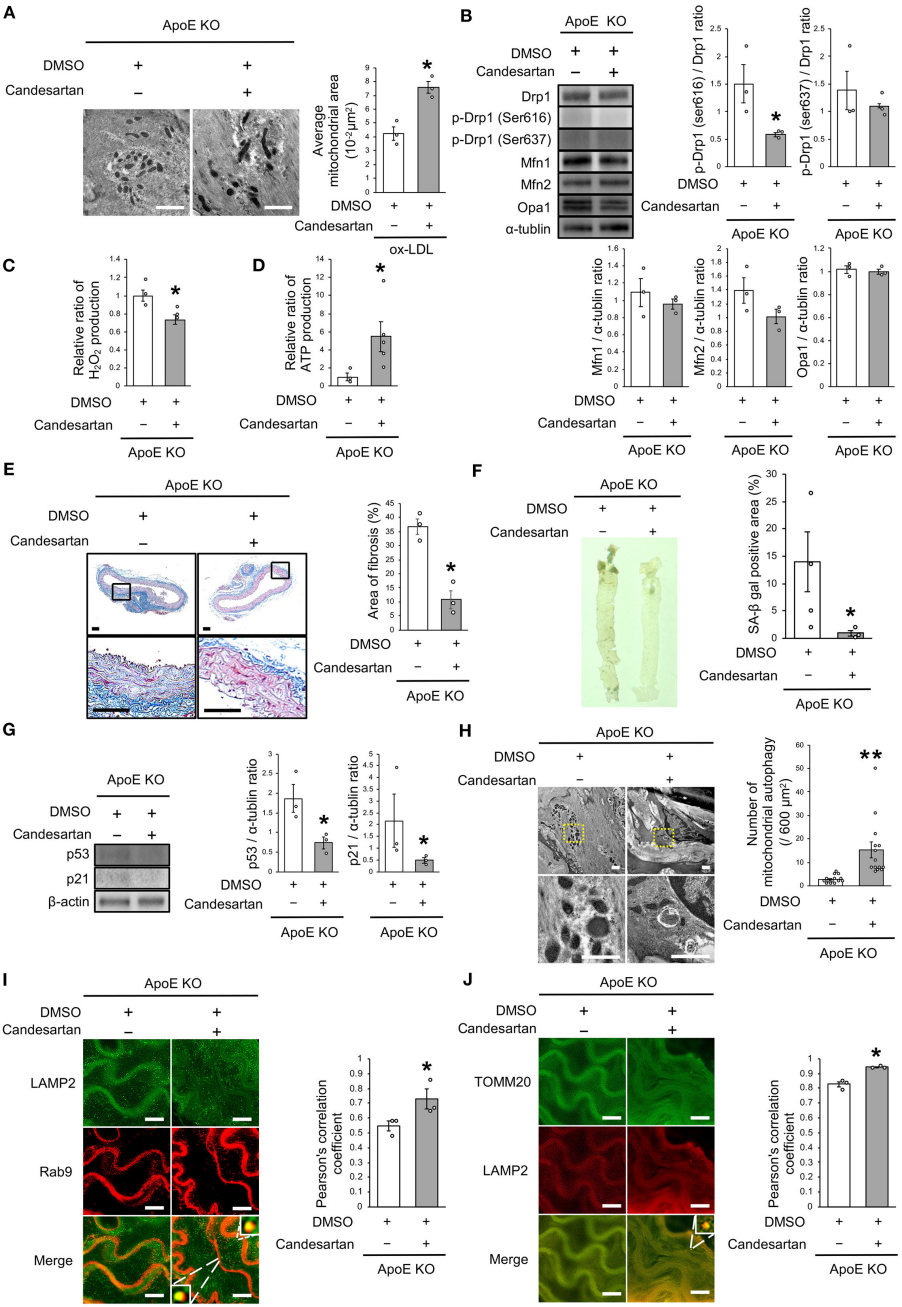
AT1R inhibition improved the imbalance of excessive mitochondrial fission and induced Rab9-dependent mitochondrial autophagy, leading to anti-vascular senescence. **(A)** Electron microscopic images and quantitative analysis of mitochondria and average mitochondrial areas in ApoE KO mice treated with Candesartan or the control. Scale bar = 1 μm. ^*^P < 0.05 vs. ApoE KO mice with the control (*n* = 3 per group). **(B)** Immunoblots and quantitative analysis for Drp1, p-Drp1 (Ser616), p-Drp1 (Ser637), Mfn1, Mfn2, Opa1, and α-tublin in ApoE KO mice treated with Candesartan or the control. ^*^P < 0.05 vs. ApoE KO mice with vehicle (*n* = 3 per group). **(C)** Relative mitochondrial H_2_O_2_ production in ApoE KO mice treated with Candesartan or the control. ^*^P < 0.05 vs. ApoE KO mice with the control (*n* = 3 per group). **(D)** Relative mitochondrial ATP production in ApoE KO mice treated with Candesartan or the control. ^*^P < 0.05 vs. ApoE KO mice with the control (ApoE KO mice with Candesartan; *n* = 5, ApoE KO mice with the control; *n* = 3). **(E)** Representative images and quantitative analysis of Masson's trichrome staining in ApoE KO mice treated with Candesartan or the control. Scale bar = 100 μm. ^*^P < 0.05 vs. ApoE KO mice with the control (*n* = 3 per group). **(F)** Representative images and quantitative analysis of SA-β gal staining of arteries in ApoE KO mice treated with Candesartan or the control. ^*^P < 0.05 vs. ApoE KO mice with the control vehicle (ApoE KO mice with Candesartan; *n* = 3, ApoE KO mice with the control; *n* = 4). **(G)** Immunoblots and quantitative analysis for p53, p21, and β-actin in ApoE KO mice with Candesartan or the control. ^*^P < 0.05 vs. ApoE KO mice with the control (*n* = 3 per group). **(H)** Electron microscopic images of mitochondrial autophagy in ApoE KO mice treated with Candesartan or the control. Scale bar = 1 μm. ^**^P < 0.01 vs. ApoE KO mice with the control (*n* = 14 per group). **(I)** Representative images of TOMM20 (green) and LAMP2 (red) immunohistochemistry in ApoE KO mice treated with Candesartan or the control. Scale bar = 10 μm. ^*^P < 0.05 vs. ApoE KO mice with the control (*n* = 3 per group). **(J)** Representative images and quantitative analysis of LAMP2 (green) and Rab9 (red) immunohistochemistry in the ApoE KO mice treated with Candesartan or the control. Scale bar = 10 μm. ^*^P < 0.05 vs. ApoE KO mice with the control (*n* = 3 per group). All Data are presented as the mean ± SEM. Statistical analysis were conducted by Wilcoxon rank sum test **(A-G,I,J)** and Student's *t*-test **(H)**.

Finally, we investigated whether AT1R inhibition attenuated vascular senescence by regulating mitochondrial dynamics and mitochondrial autophagy in ApoE KO mice. Food intake during the experiment, body weight, heart rate, and blood pressure were not different between ApoE KO mice treated with candesartan or the control vehicle (Table 3). EM revealed that excessive mitochondrial fission was reduced in ApoE KO mice treated with candesartan when compared to that in the control mice (Figure 8A). The arterial expression of Drp1 phosphorylation at serine 616 was lower in ApoE KO mice treated with candesartan when compared to that in the control mice. Other mitochondrial dynamics factors were not significantly different between the two groups (Figure 8B). H_2_O_2_ production was lower and ATP production was higher in ApoE KO mice administered candesartan (Figures 8C,D). The ratio of arterial fibrosis and the senescence area were also lower in ApoE KO mice administered with candesartan when compared with the control (Figures 8E,F). Immunoblot analysis showed that the expression of p53 and p21 was lower in ApoE KO mice treated with candesartan (Figure 8G). To examine mitochondrial autophagy induced by AT1R inhibition, we performed EM and immunohistochemistry. The number of autophagy-engulfing mitochondria was higher in ApoE KO mice administered with candesartan when compared to the control (Figure 8H). The colocalization of LAMP2 with Rab9 or TOMM20 was also higher in ApoE KO mice treated with the ARB when compared with the control (Figures 8I,J). Taken together, AT1R inhibition was found to contribute to improved mitochondrial quality control through the suppression of excessive mitochondrial fission and the induction of Rab9-dependent mitochondrial autophagy, causing anti-arterial senescence in ApoE KO mice.

**Table 3:** Body weight, food intake, heart rate, and blood pressure of ApoE KO mice administered with Candesartan or the control vehicle.

	**ApoE KO + control vehicle (*n* = 13)**	**ApoE KO + Candesartan (*n* = 14)**	***P*** **-value**
BW (g)	29.3 ± 1.7	28.7 ± 2.5	N.S.
FI (g/day)	3.9 ± 0.2	4.0 ± 0.3	N.S.
HR (bpm)	680 ± 41	707 ± 15	N.S.
BP (mmHg)	117 ± 17/77 ± 13	113 ± 13/74 ± 17	N.S.

*All values are presented as the mean and SD. BW, body weight; FI, food intake; HR, heart rate; BP, blood pressure*.

## Discussion

Our findings indicate that AT1R inhibition attenuates excessive mitochondrial fission that is induced by ox-LDL and thus augments mitochondrial autophagy, which is crucial for maintaining mitochondrial quality control and the effect of anti-senescence. In human VSMCs exposed to ox-LDL, AT1R inhibition suppresses Drp1 activation through the CRAF/MEK/ERK pathway, which is downstream of the AT1R signal, and did not induce conventional *Atg7*/LC3-dependent autophagy but Rab9-dependent alternative autophagy. The administration of candesartan to ApoE KO mice retarded excessive mitochondrial fission and induced Rab9-dependent alternative autophagy, resulting in anti-vascular senescence.

Regardless of mitochondrial dynamics, there were some reports of mitochondrial fission induced by ox-LDL or a high-fat diet in various cells or animals. Treatment with ox-LDL induces excessive mitochondrial fission in rat annulus fibrosus cells (24) and the hearts of high-fat-fed C57BL/6 mice showed mitochondrial fission (17). As shown in Figure 4, ox-LDL activated CRAF, MEK, and ERK. Either inhibition of CRAF, MEK, or ERK inactivated the phosphorylation of Drp1 at serine 616 induced by ox-LDL. Furthermore, AT1R inhibition suppressed the activation of CRAF, MEK, ERK, and Drp1 induced by ox-LDL. These results revealed that ox-LDL activated Drp1 through AT1R and CRAF/MEK/ERK pathway. It has been reported that Drp1 may be involved in this cascade in various cells. Ang II activates Drp1 through MEK/ERK signaling by interacting with protein kinase Cδ in rat VSMCs (25). Another study has reported that the high-mobility group box-1 activated phosphorylation of Drp1 at serine 616 through ERK in rat pulmonary artery SMCs (26). Ox-LDL was also reported to activate RAF/MEK/ERK, one of the most well-defined pathways in cancer biology, in rat VSMCs (27, 28). The signals indicated by these reports are consistent with the cascade of Drp1 activation in human VSMCs. As mentioned previously, ox-LDL upregulated ERK through receptor association with LOX-1 and AT1R (9). However, it had not been previously reported that AT1R and its downstream signal are involved in ox-LDL-induced mitochondrial fission, as we revealed in this investigation. Furthermore, there are no previous reports indicating that the association of LOX-1 and AT1R plays a role in regulating mitochondrial dynamics.

Many researchers have reported that mitochondrial fission adversely affects atherosclerosis, various cell types, and diseases. Drp1 inhibition attenuates VSMC proliferation and reduces ROS production (25). Anoxia reoxygenation activated phosphorylation of Drp1 at serine 616, which facilitated cardiomyocyte death in HL-1 cells, but was attenuated by the inhibition of cyclin dependent kinase 1 and protein kinase Cδ (29). HIF-1α activation in human pulmonary artery SMCs leads to mitochondrial fission by cyclin B1/CDK1-dependent phosphorylation of Drp1 at serine 616, resulting in pulmonary artery remodeling (30). Conversely, there are some reports that suppressing mitochondrial fission improves mitochondrial conditions. Prior to the administration of mdivi-1 to an ischemia reperfusion murine model, it had inhibited mitochondrial fission, which prevented the opening of the mitochondrial permeability transition pore and decreased infarct size (31). However, excessive suppression of fission has been reported as an adverse effect. Cardiomyocyte-specific ablation of Drp1 mice resulted in the accumulation of damaged mitochondria and left ventricular dysfunction, and finally death within 13 weeks. Under ischemia reperfusion conditions, mice treated with a high dose of mdivi-1 showed a greater degree of myocardial damage than the control mice (32). This is because excessive inhibition of mitochondrial fission suppresses mitochondrial autophagy, leading to the accumulation of damaged mitochondria. Indeed, we also found that ox-LDL treated with a high concentration of mdivi-1 increased p53 expression when compared to that in the control (data not shown). Given these results, it may be important to maintain an optimal balance between mitochondrial fission and fusion.

There have been some reports on the relationship between RAS signaling and autophagy in various cells and animals. However, all these studies have shown that Ang II induced conventional autophagy and its inhibition by AT1R inhibition provided beneficial protective effects on the cardiovascular system. In addition, there have been no reports examining the relationship between RAS signaling and alternative autophagy. Ang II increased LC3-II expression in rat VSMCs, resulting in decreased apoptosis (33). Another study revealed that Ang II induced LC3-depenent autophagy through AT1R/RhoA/Rho kinase, leading to hypertrophy, and ARB reduced autophagy (34). In human umbilical vein endothelial cells, Ang II induces *Atg5*-dependent autophagy via AT1R and decreases nitric oxide production, leading to dysfunction of human umbilical vein endothelial cells (35). For mitochondrial autophagy, one study reported that the renovascular hypertension model for domestic pigs improved left ventricular hypertrophy by reducing the myocardial LC3-II/LC3-I ratio and colocalization of Parkin and Tomm20, suggesting that ARB suppressed LC3-dependent mitochondrial autophagy (36).

There are two forms of autophagy, as mentioned above: LC3-dependent autophagy and Rab9-dependent autophagy (21, 23, 37). Since we have recently demonstrated that Rab9-dependent alternative autophagy causes estrogen-mediated mitochondrial autophagy through the SIRT1/LKB1/AMPK/Ulk1 pathway (21), we examined which type of autophagy AT1R-induced mitochondrial autophagy contributes to. As shown in Figure 5, the colocalization of LAMP2-Rab9 signals and the number of autophagosome-engulfed mitochondria in VSMCs was increased by AT1R inhibition treated with ox-LDL. It is noteworthy that this phenotype was affected by the knockdown of Rab9 and was not diminished after the silencing of *Atg7*, which is essential for inducing LC3-dependent conventional autophagy, suggesting that AT1R inhibition in VSMCs exposed to ox-LDL induces *Atg7*-independent and Rab9-dependent alternative autophagy. One study reported that ox-LDL treatment increased with the colocalization of LC3-TOMM20 and this colocalization was decreased by 3-methyladenine treatment, an inhibitor of conventional autophagy, suggesting that ox-LDL induced LC3-dependent mitochondrial autophagy. However, ox-LDL reduces mitochondrial function and generates ROS. This is because mitochondria impaired by ox-LDL cannot be eliminated by ox-LDL-mediated physiological mitochondrial autophagy (38). Since AT1R inhibition attenuated mitochondrial dysfunction and ROS production, AT1R inhibition regulated mitochondrial autophagy derived from Rab9-dependent alternative autophagy is crucial to sustain mitochondrial quality control in VSMCs.

Although it is generally believed that mitochondrial autophagy requires fission, we revealed that AT1R inhibition simultaneously induces mitochondrial fusion and mitochondrial autophagy. This is because AT1R inhibition did not physiologically suppress mitochondrial fission in our experiment. Indeed, as assessed by EM and Mitotracker, AT1R inhibition did not force the proportion of the cells to have excessive mitochondrial fusion, but decreased the number of cells with excessive mitochondrial fission induced by ox-LDL. Moreover, it has been reported that the amount of mitochondrial autophagy decreases with hepatocyte-specific deletion in Drp1 mice, whereas mitochondrial autophagy occurs in Drp1-Opa1 double knockout mice, indicating that fission is not necessarily required for the induction of mitochondrial autophagy (39). A previous study reported that either statin or ARB had a mild inhibitory effect on LOX-1 expression, and the combination of statin and ARB inhibited LOX-1 completely in the arteries of ApoE KO mice fed a high fat diet (10). Given this report and our findings, AT1R inhibition using ARB is the potential key therapy and combined use of statin with ARB could facilitate to improve residual risk from lipid-lowering therapies through mitochondrial quality control in CVD patients.

**Figure 9:**
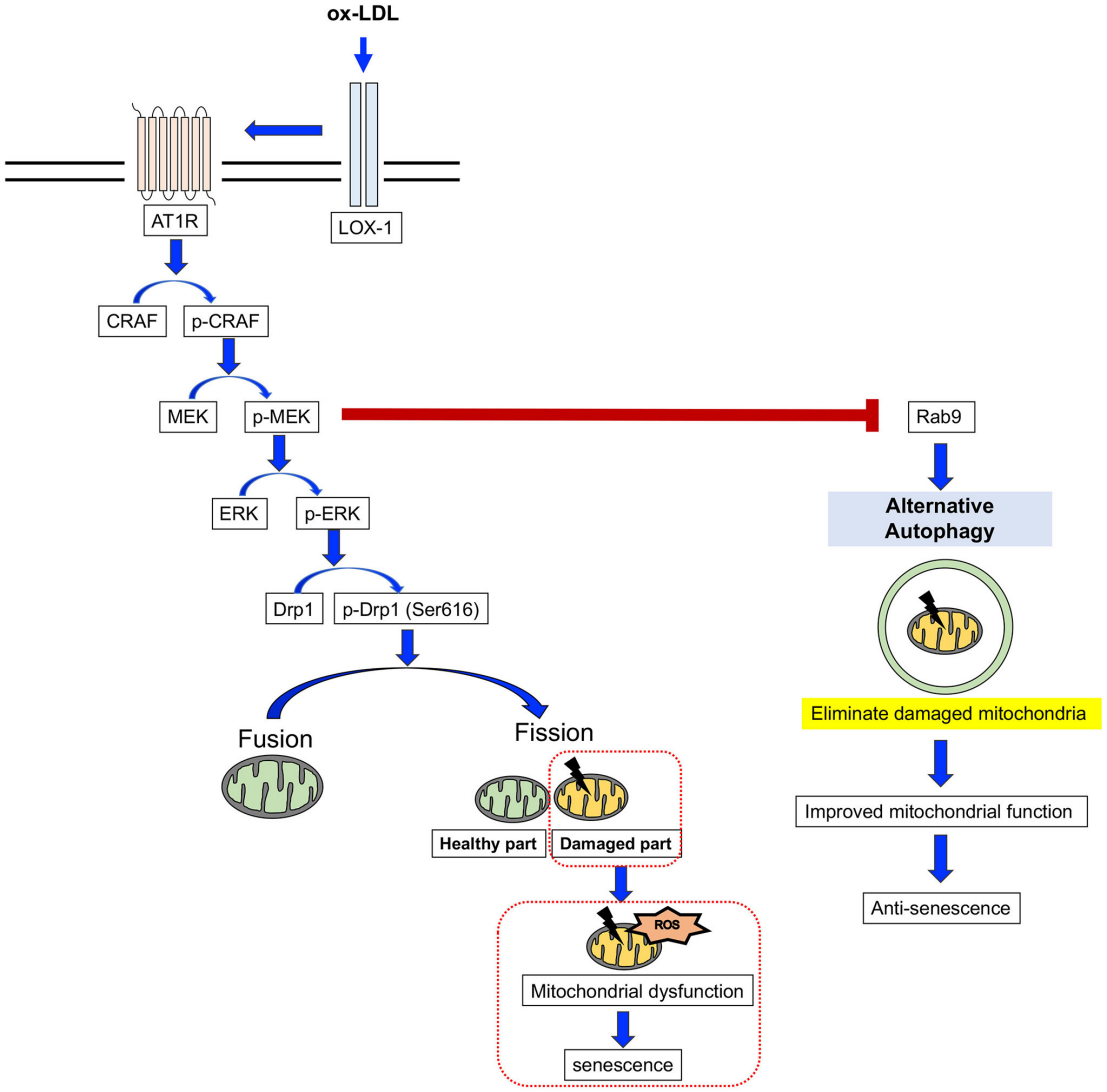
The mechanism for the AT1R inhibition of anti-senescence through mitochondrial quality control. ox-LDL induced mitochondrial fission through the association of LOX-1 and AT1R and the CRAF/MEK/ERK pathway, resulting in mitochondrial dysfunction and senescence. AT1R inhibition attenuated senescence by suppression of this cascade and induced Rab9-dependent alternative autophagy through the CRAF/MEK axis and aspects of the mitochondrial quality control process in human VSMCs.

Here, we have revealed for the first time that AT1R inhibition attenuates cellular senescence by regulating mitochondrial dynamics and inducing Rab9-dependent alternative autophagy through the CRAF/MEK/ERK axis in terms of mitochondrial quality control in human VSMCs (Figure 9). We hope that these findings will help to retard vascular senescence and contribute to the development of a method by which to reduce residual risk of CVD.

### Limitations

Despite the promising nature of our results, there were some acknowledged limitations in this study. First, we did not conduct *in vivo* experiments for arterial-specific loss of function of Rab9 or Drp1 conditional knockout mice. Second, we did not determine the precise mechanisms of mitochondrial autophagy induced by AT1R inhibition. In particular, in the future we should aim to determine how Rab9-dependent alternative autophagy recognizes impaired mitochondria in a PINK1-Parkin pathway-independent manner. Further studies are thus required to confirm the role of mitochondrial autophagy.

## Data Availability Statement

The raw data supporting the conclusions of this article will be made available by the authors, without undue reservation.

## Ethics Statement

The animal study was reviewed and approved by The Faculty of Medicine at Kagoshima University.

## Author Contributions

YU performed most of the experiments, analyzed the data, interpreted the results, and wrote the manuscript. YI designed the study, interpreted the results, and edited the manuscript. YS and MI performed the experiments and analyzed the data. YA provided project resources. MO designed and supervised the study, interpreted the results, and provided the project resources. All authors have approved the submitted version of the manuscript.

## Funding

This work was supported by the Japan Society for the Promotion of Science KAKENHI (Grant Nos. JP19K07891 and JP21K15649).

## Conflict of Interest

YI is a member of the Editorial Team of *Frontiers in Cardiovascular Medicine*, and MO is a member of the Editorial Team of *Circulation Journal*. The remaining authors declare that the research was conducted in the absence of any commercial or financial relationships that could be construed as a potential conflict of interest.

## Publisher's Note

All claims expressed in this article are solely those of the authors and do not necessarily represent those of their affiliated organizations, or those of the publisher, the editors and the reviewers. Any product that may be evaluated in this article, or claim that may be made by its manufacturer, is not guaranteed or endorsed by the publisher.

## Abbreviations

LOX-1: lectin-like oxidized low-density lipoprotein scavenger receptor-1
AT1R: angiotensin II type 1 receptor
VSMCs: vascular smooth muscle cells
ApoE KO: apolipoprotein E deficient
ox-LDL: oxidized low-density lipoprotein
CVD: cardiovascular disease
ROS: reactive oxygen species
ATP: adenosine triphosphate
Drp1: dynamin-related protein 1
Mfn1: mitofusin 1
Mfn2: mitofusin 2
Opa1: Optic-atrophy 1
Candesartan: Candesartan cilexetil
DMSO: dimethyl sulfoxide
SA-β gal: Senescence-associated β-galactosidase
SEM: standard error of the mean
ARB: angiotensin II type 1 receptor blocker
Ang II: angiotensin II
EM: electron microscopy
RAS: renin-angiotensin system
EBA: Extraction Buffer A
PBS: phosphate-buffered saline.
