# Supplementary material for: Association of Lectin-Like Oxidized Low-Density Lipoprotein Receptor-1 With Angiotensin II Type 1 Receptor Impacts Mitochondrial Quality Control, Offering Promise for the Treatment of Vascular Senescence
Source: Front Cardiovasc Med. 2021 Nov 17;8:788655. doi: 10.3389/fcvm.2021.788655 (PMC8637926; doi:10.3389/fcvm.2021.788655)

Supplemental Figure 1

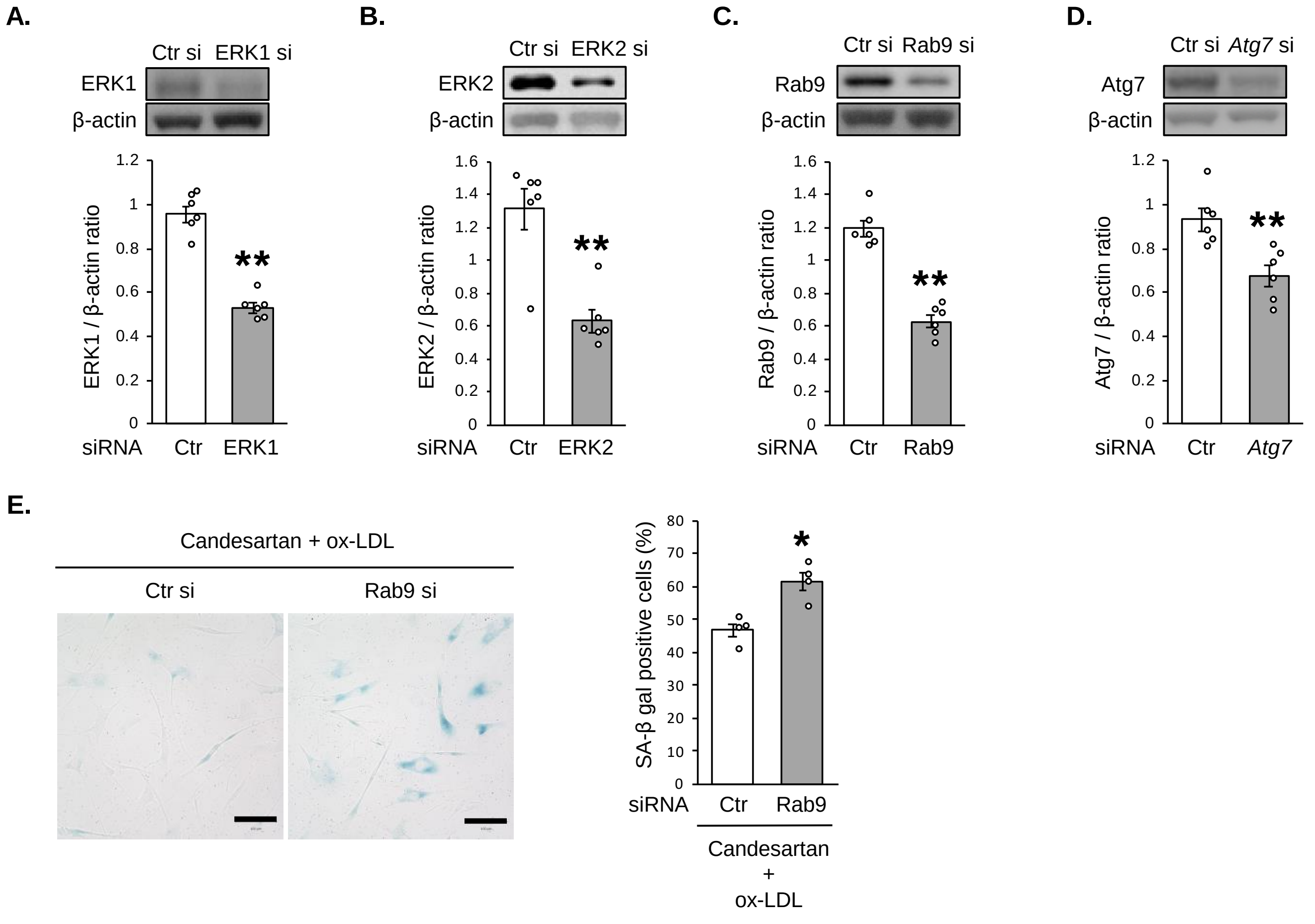

Supplemental Figure 2

Figure1-B

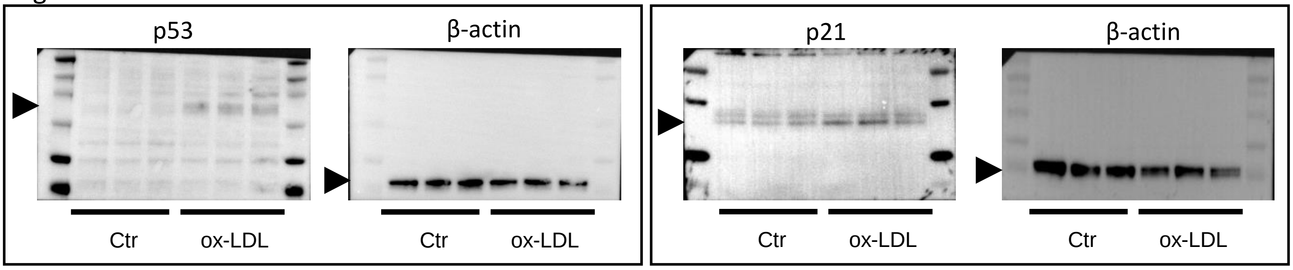

Figure1-I

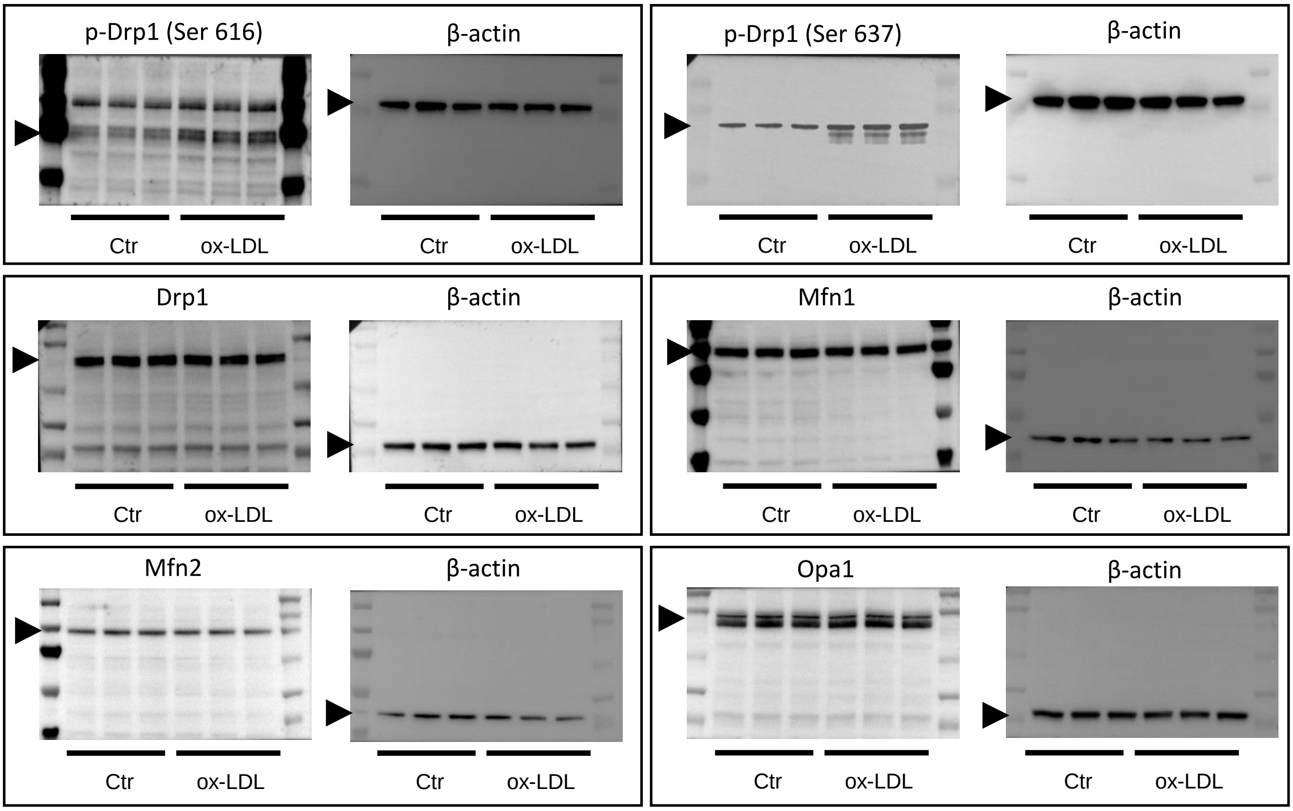

Figure1-J

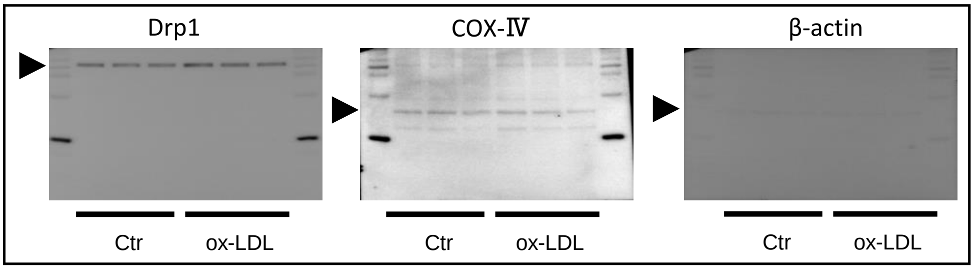

Figure2-C

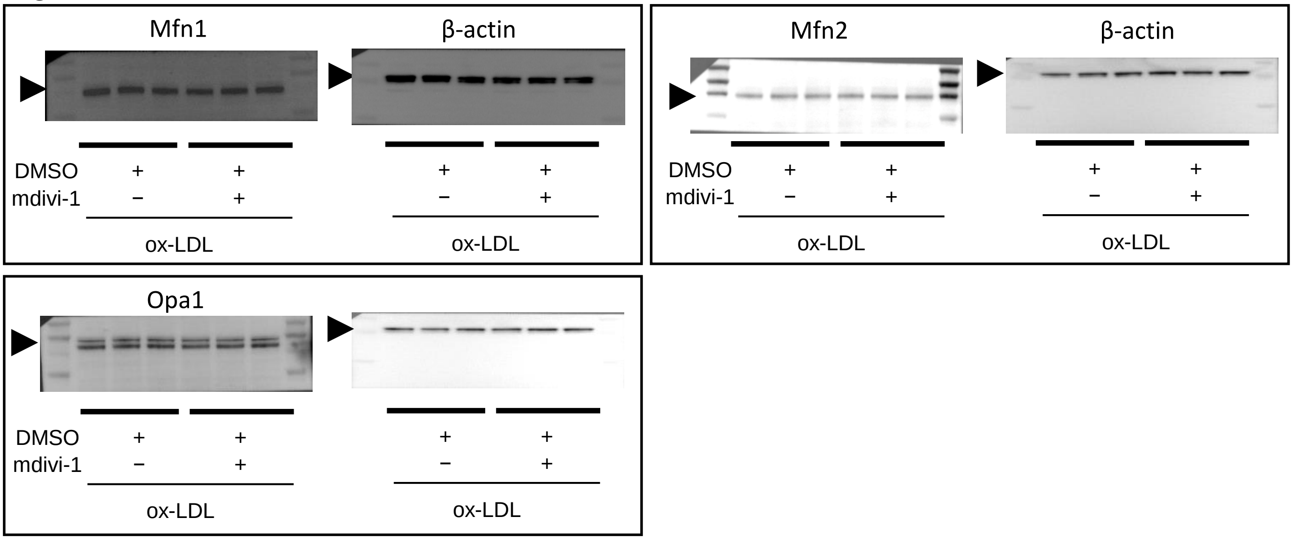

Supplemental Figure 2

Figure2-E

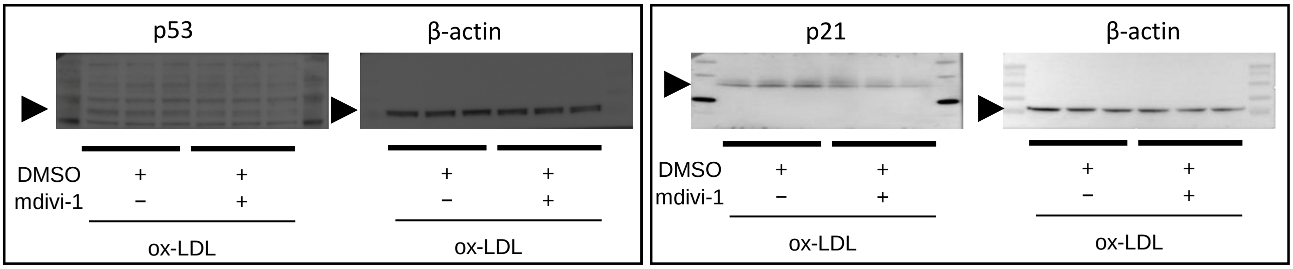

Figure3-C

※Cande; Candesartan

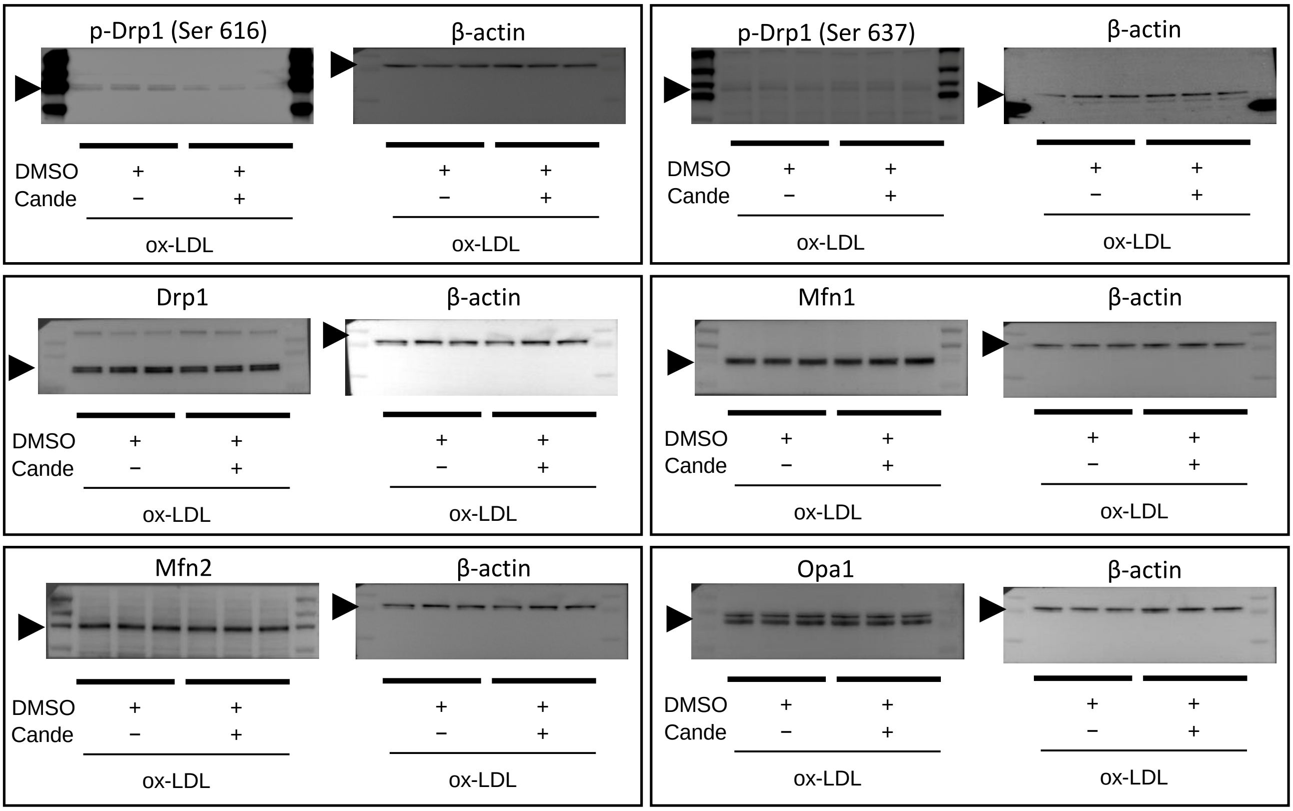

Figure3-I

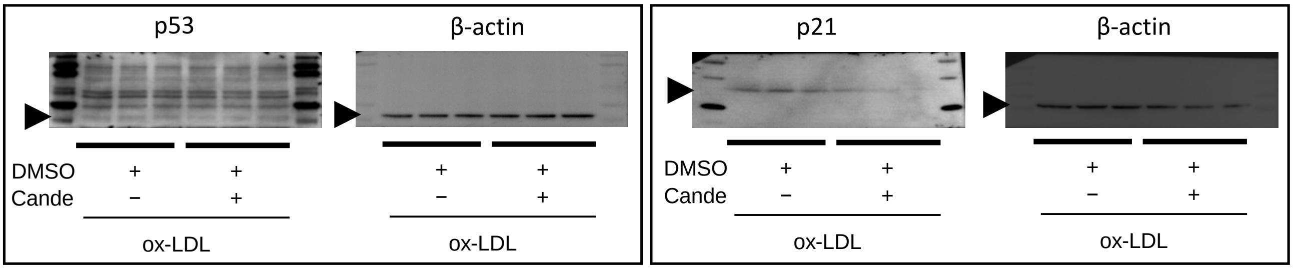

Figure4-A

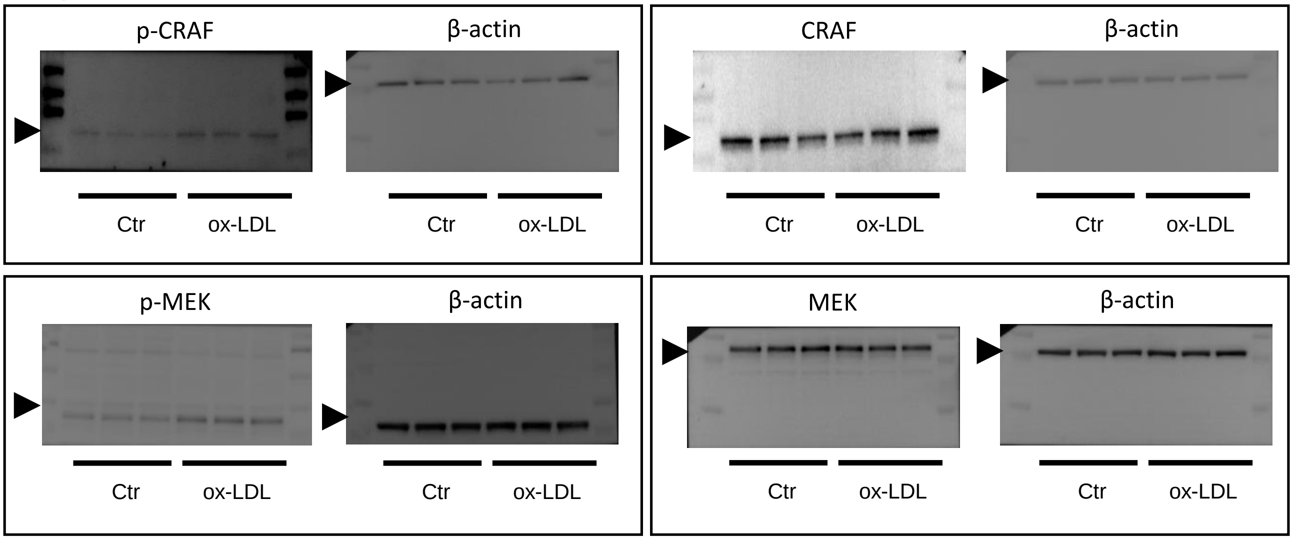

Supplemental Figure 2

Figure4-A

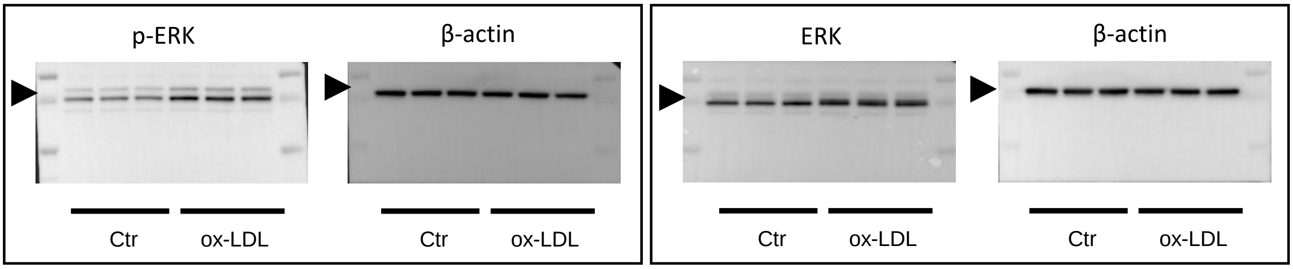

Figure4-B

※Dabra; Dabrafenib

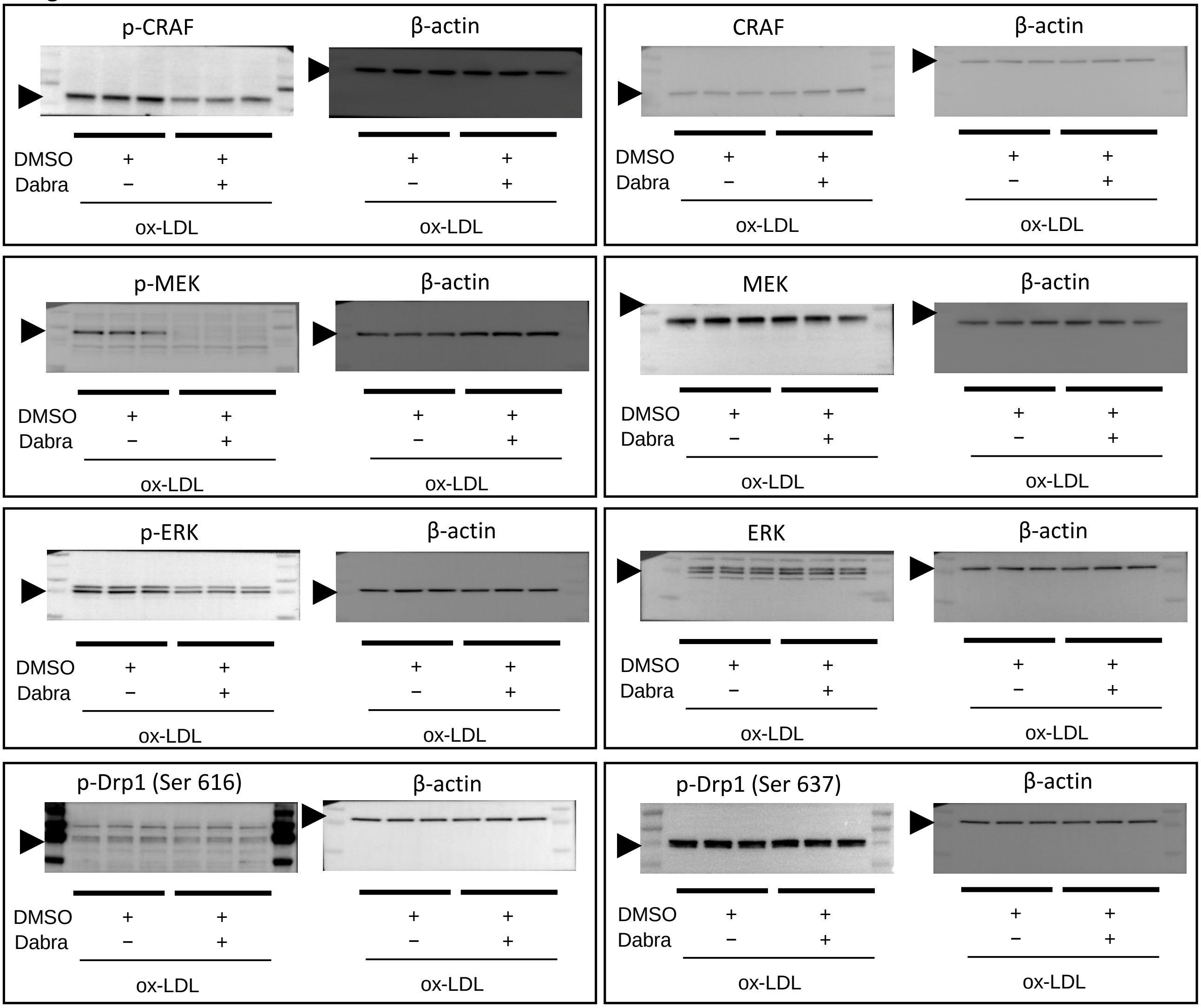

Figure4-C

※PD; PD184325

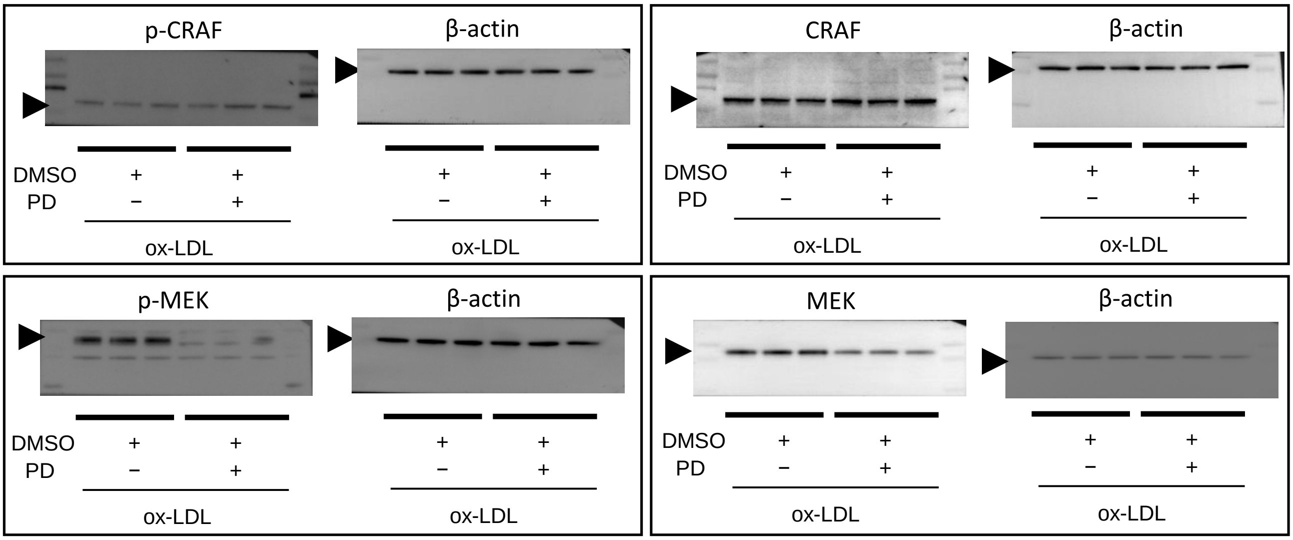

Supplemental Figure 2

Figure4-C

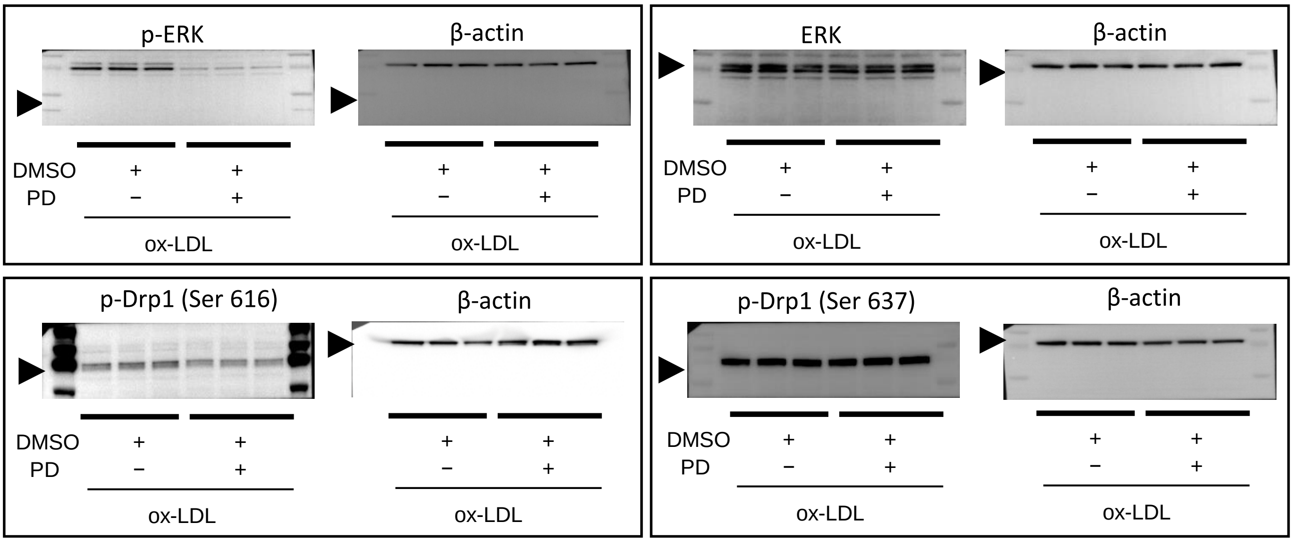

Figure4-D ※SCH; SCH772984

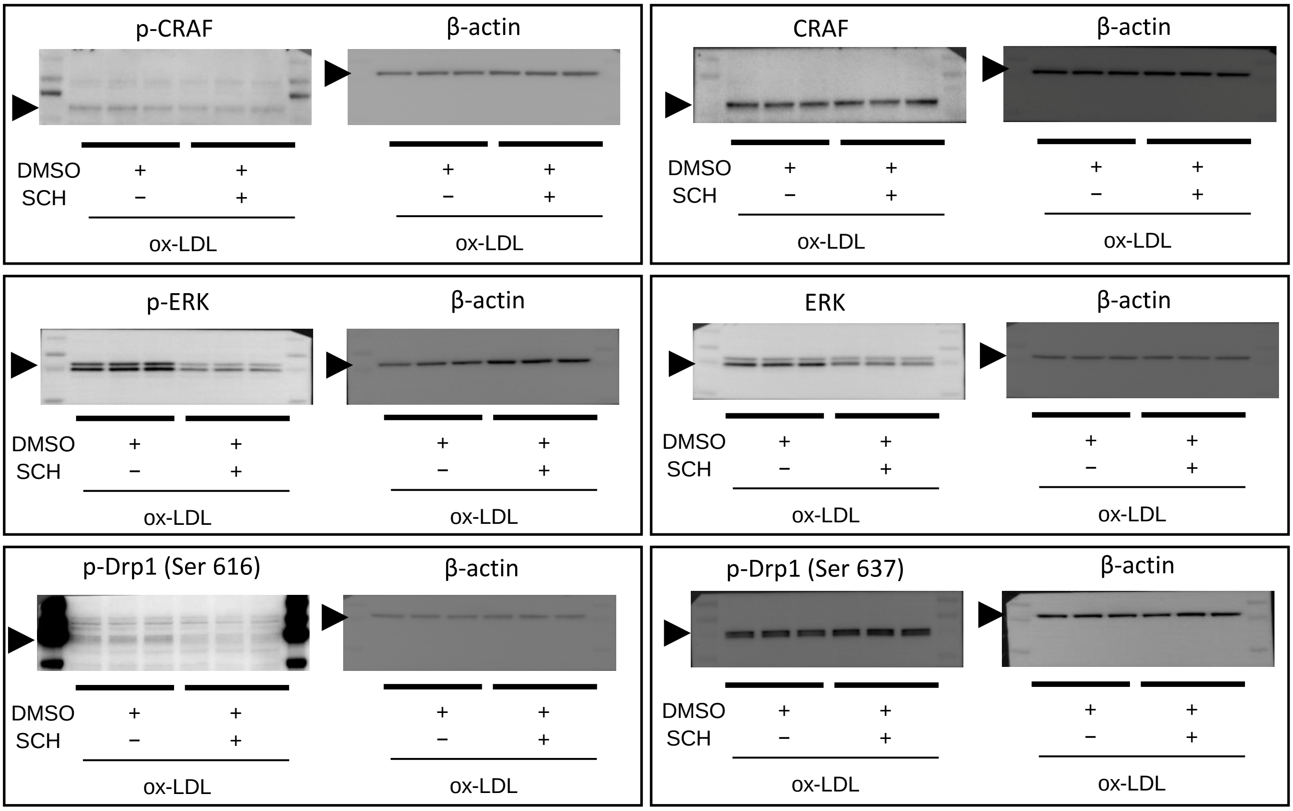

Figure4-E

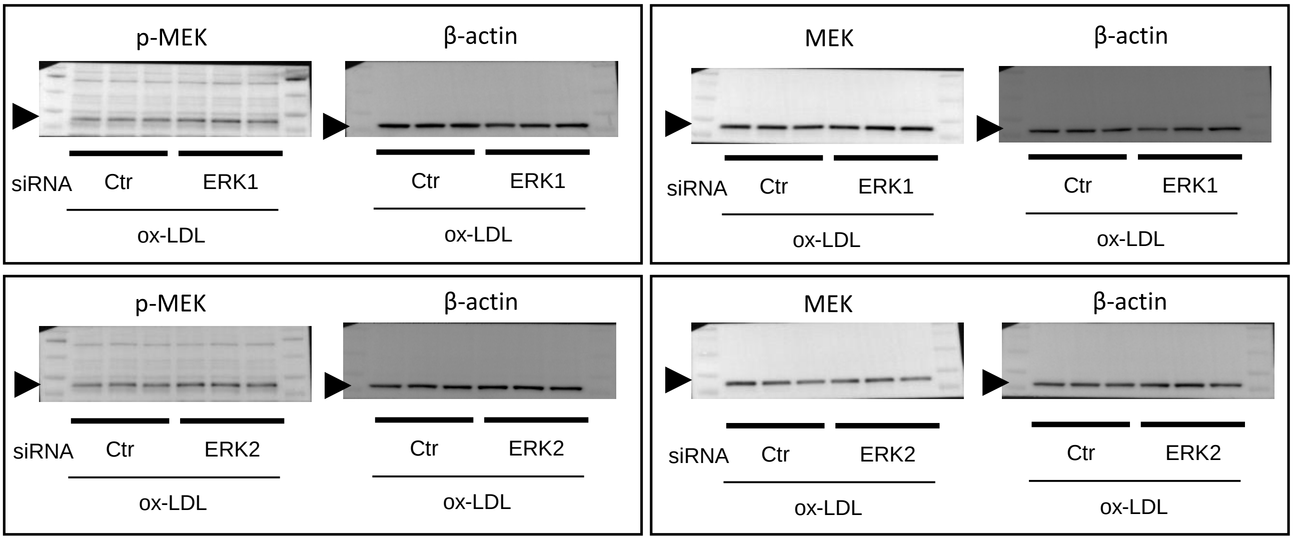

Supplemental Figure 2

Figure4-F

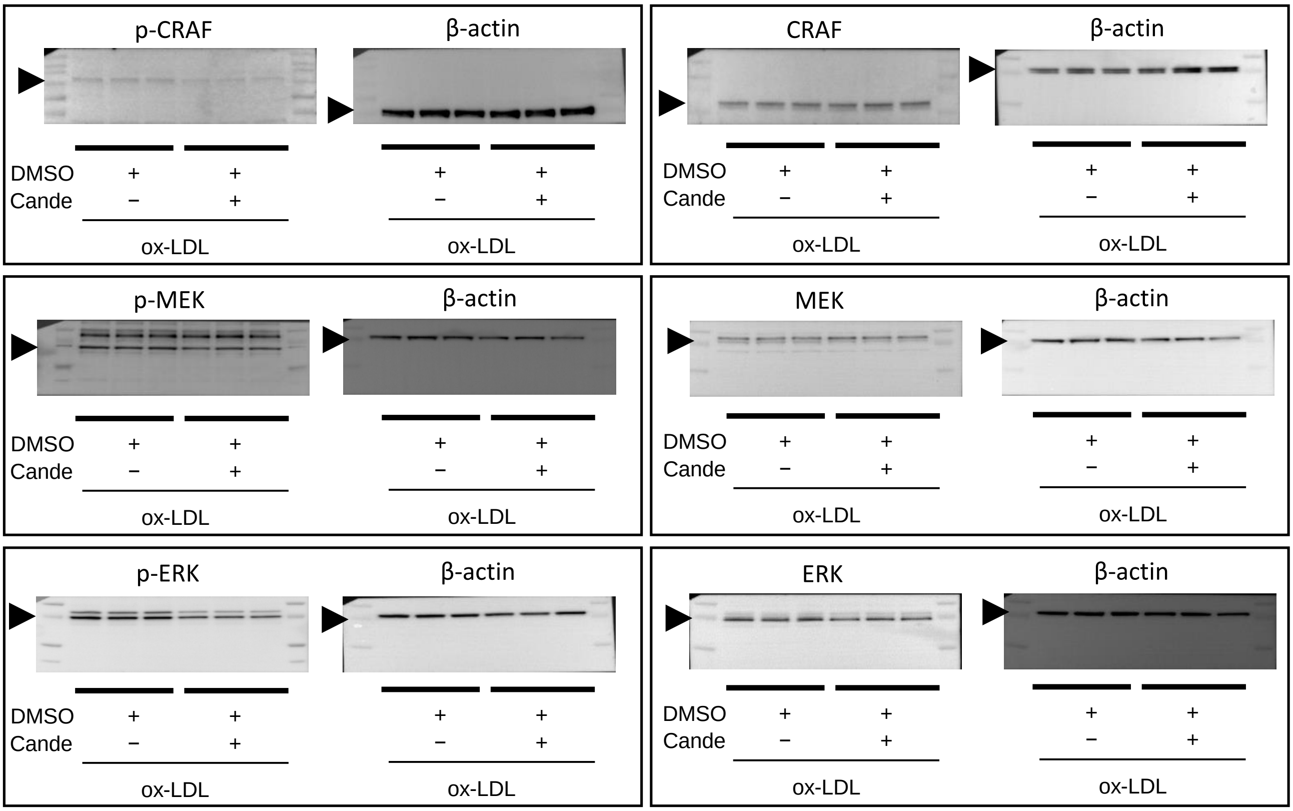

Figure7-B

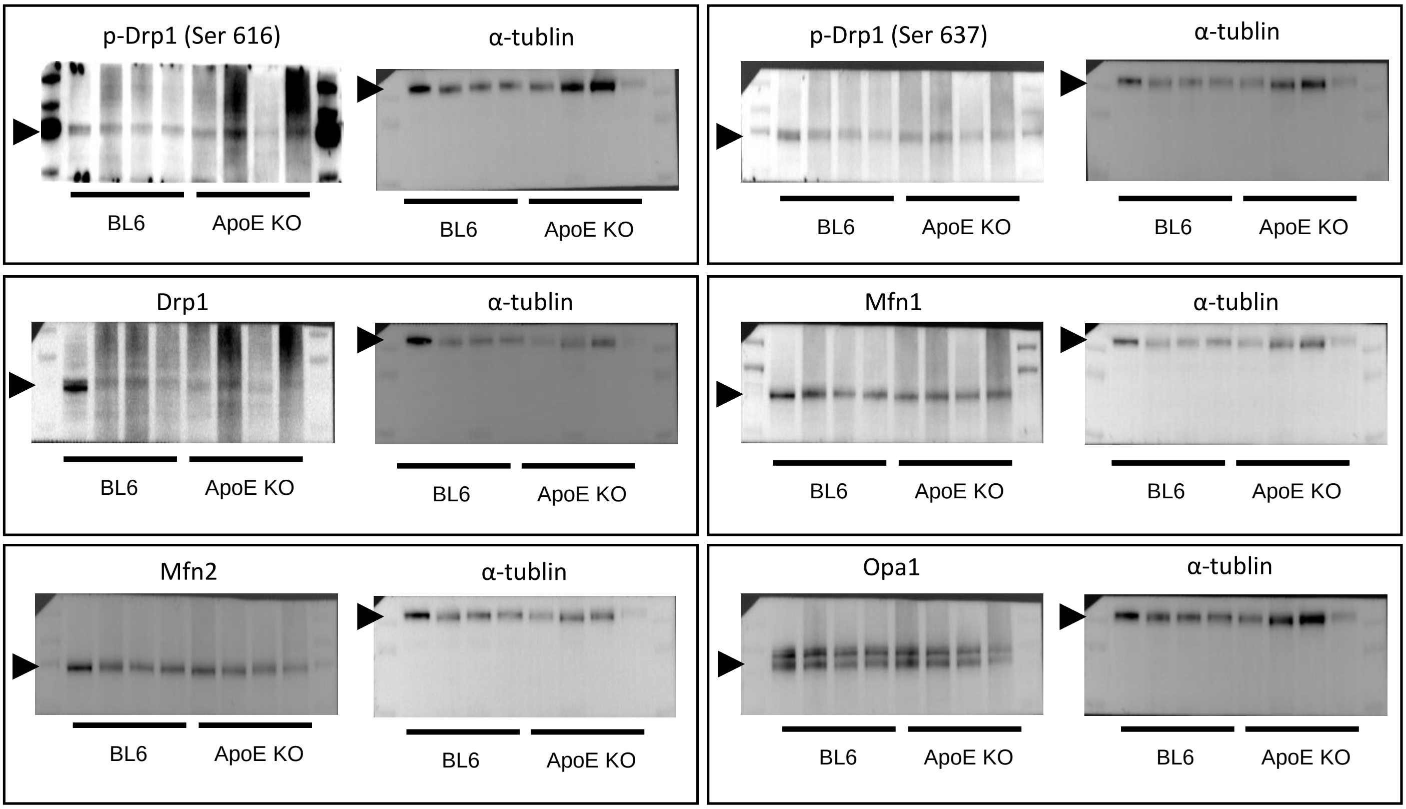

Figure7-G

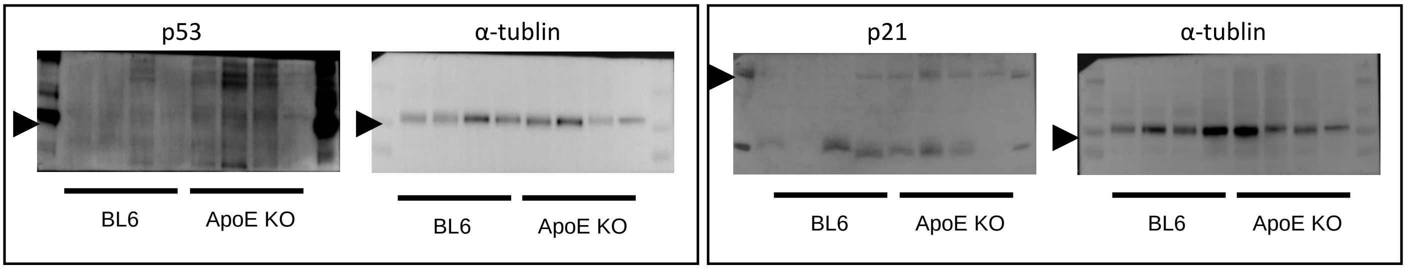

Supplemental Figure 2

Figure7-I

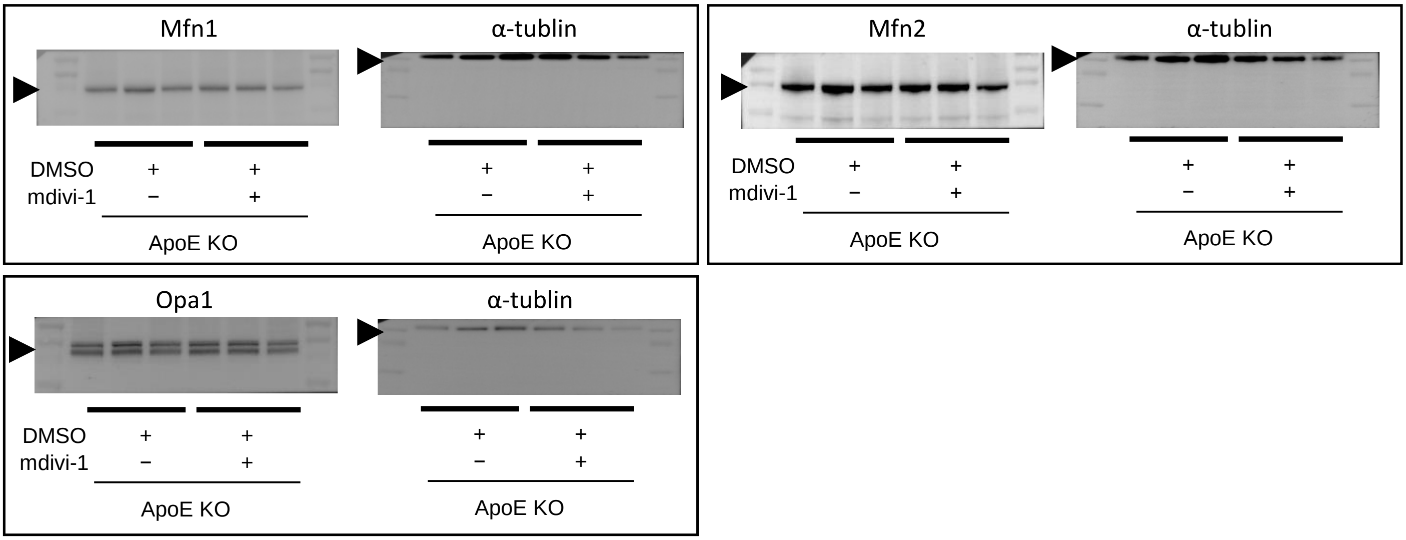

Figure8-B

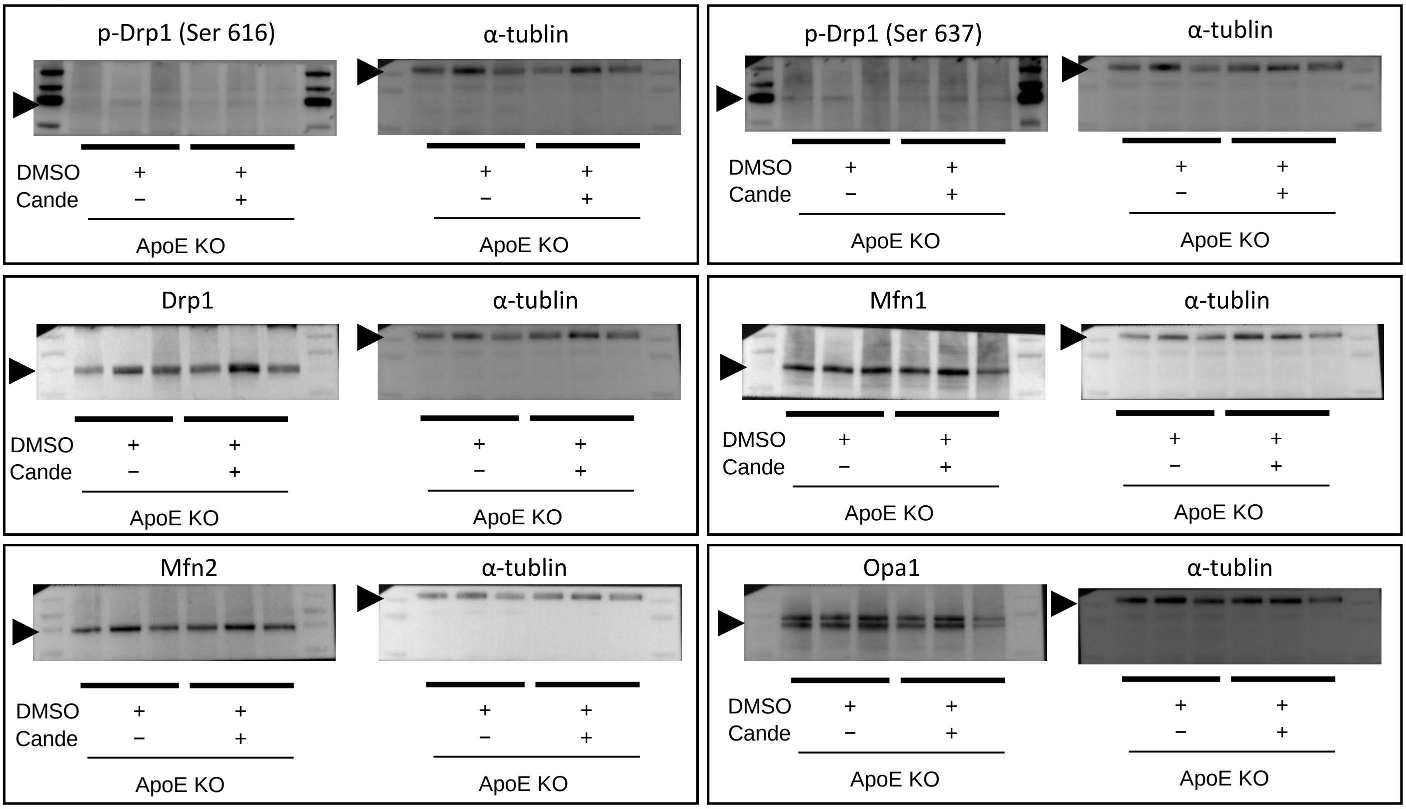

Figure8-G

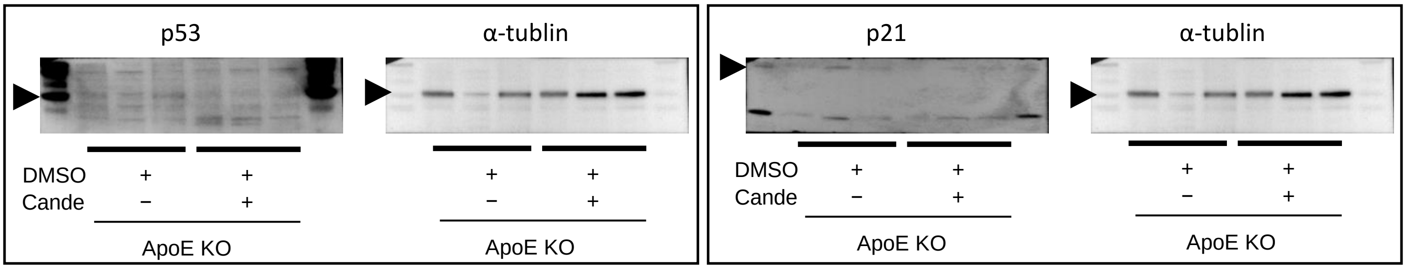

Supplemental Figure 1

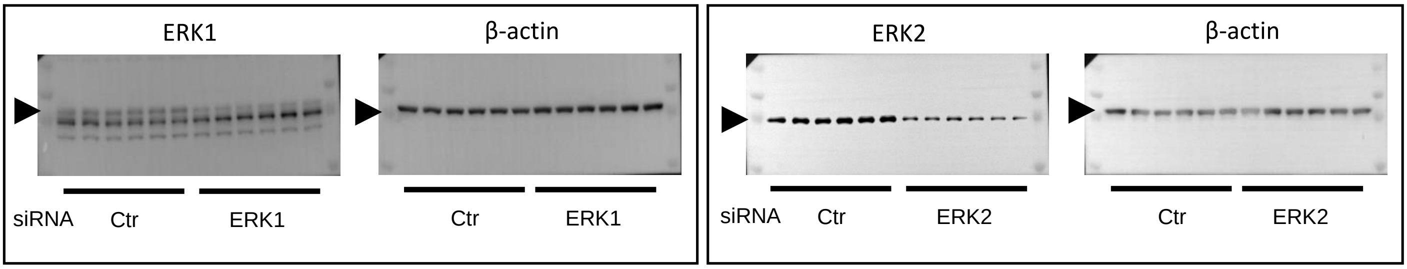

Supplemental Figure 2

Supplemental Figure 1

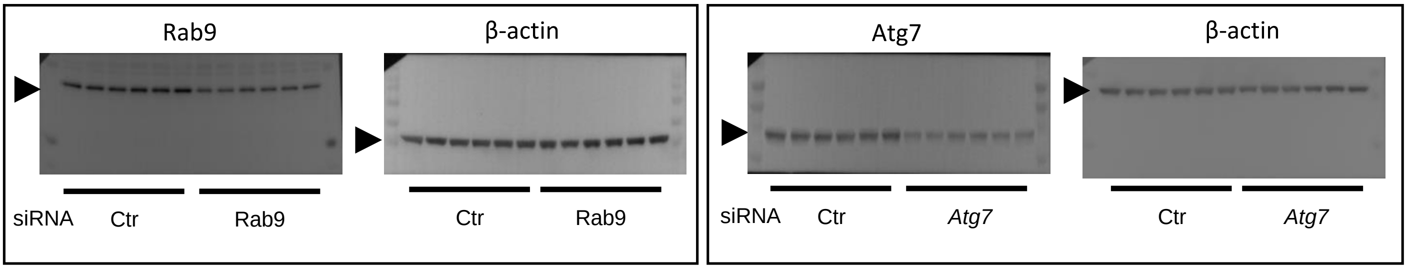

Supplement: Supplementary file 1 [file Data_Sheet_1.PDF]
